# Diffusion control in biochemical specificity

**DOI:** 10.1016/j.bpj.2022.03.005

**Published:** 2022-03-09

**Authors:** Jose L. Alejo, Christopher P. Kempes, Katarzyna P. Adamala

**Affiliations:** 1 Department of Genetics, Cell Biology and Development, University of Minnesota, Minneapolis, Minnesota; 2 The Santa Fe Institute, Santa Fe, New Mexico

## Abstract

Biochemical specificity is critical in enzyme function, evolution, and engineering. Here we employ an established kinetic model to dissect the effects of reactant geometry and diffusion on product formation speed and accuracy in the presence of cognate (correct) and near-cognate (incorrect) substrates. Using this steady-state model for spherical geometries, we find that, for distinct kinetic regimes, the speed and accuracy of the reactions are optimized on different regions of the geometric landscape. From this model we deduce that accuracy can be strongly dependent on reactant geometric properties even for chemically limited reactions. Notably, substrates with a specific geometry and reactivity can be discriminated by the enzyme with higher efficacy than others through purely diffusive effects. For similar cognate and near-cognate substrate geometries (as is the case for polymerases or the ribosome), we observe that speed and accuracy are maximized in opposing regions of the geometric landscape. We also show that, in relevant environments, diffusive effects on accuracy can be substantial even far from extreme kinetic conditions. Finally, we find how reactant chemical discrimination and diffusion can be related to simultaneously optimize steady-state flux and accuracy. These results highlight how diffusion and geometry can be employed to enhance reaction speed and discrimination, and similarly how they impose fundamental restraints on these quantities.

## Significance

Biochemical reactions require high specificity in discriminating between similar substrates. Using established chemical kinetic modeling, we explore the role of reactant geometries and diffusion in this specificity. Our results demonstrate that accuracy can be dependent on geometric/diffusive properties even when the underlying reactions are limited by their chemical processes. We show how diffusion can optimally discriminate specific geometry-reactivity combinations of incorrect substrates, as well as the substantial effects of diffusion on specificity in relevant biological scenarios. For similar correct and incorrect substrate geometries (as is the case for polymerases or the ribosome), speed and specificity are maximized in opposing regions of the geometric landscape. Finally, we derive relations between reactivity and diffusion for the simultaneous optimization of speed and accuracy.

## Introduction

It has long been understood that reaction kinetics can be affected by the geometry and diffusion of the participating reactants (1, 2, 3, 4). Diffusion is an essential attribute of much of chemistry and biology, imposing fundamental limits on the transport processes of molecular reactants. An aspect of this spatial diffusion that has not been as researched is its effect on enzyme-substrate specificity. Enzymes can engage correct or incorrect substrates and consequently catalyze the formation of distinct products (5,6). High levels of specificity are therefore common in biochemical reactions, and essential in central dogma polymerization processes (7, 8, 9).

The accuracy of these enzyme reactions can be quantified by comparing the formation rates of the correct and incorrect products and can be understood in terms of the underlying chemical kinetics (10, 11, 12, 13, 14). The basic mechanisms of this substrate discrimination are centered on binding, recognition, and catalysis (15,16), although numerous more complex discrimination mechanisms exist beyond these basic categories, such as catalytic site gating (17, 18, 19), kinetic proofreading (20,21), and subsequent quality control (22,23). In an idealized reaction, reactant diffusion entails an additional discrimination mechanism that is implicit in the binding kinetics and independent of the recognition and catalysis stages. These spatial diffusion kinetics are determined by factors such as reactant geometry and reactive sites (24, 25), solvent properties (26), molecular crowding (4,27,28), and intermolecular forces (2,29). Successful reactions rates will therefore be limited by the time it takes to achieve the proximity and orientations required for binding and subsequent catalysis. Under these assumptions, the impact of spatial diffusion on speed and accuracy will depend on how its rates relate to the chemical kinetics that follow.

In this study, we investigate geometric and diffusive effects on the speed and accuracy of promiscuous chemical reactions. Initially, we establish the classical quantitative expressions for rate and accuracy in steady state (10,30). As a general reactant geometric model, we employ the previously characterized framework of asymmetrically reactive spheres (24,31) with varying sizes, reactive sites, and chemical reaction rates. We find that, in specific kinetic regimes, speed and accuracy are optimized in different regions of the geometric landscape (reactant and reactive site sizes). For reactions where the cognate and near-cognate sizes are different, we find the enzyme can discriminate optimally substrates with a specific geometry and reactivity. Alternatively, when the substrates have identical geometries, speed and accuracy are optimized in opposing regions of the geometric landscape. We also apply recently developed expressions (32) to relate chemical and diffusive discrimination and optimize steady-state total flux and accuracy. Finally, we establish that, in biochemically relevant environments, diffusive effects on accuracy (specifically for protein synthesis) can be considerable even far from kinetic extremes. The results derived are an initial approximation to the exploration of wide-ranging relations between chemistry, geometry, diffusion, and accuracy.

## Methods

### General expressions

**Figure 1:**
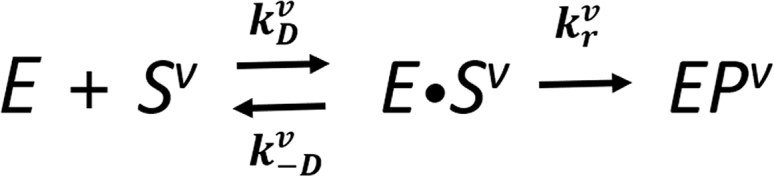
Basic scheme of enzyme-substrate diffusion and reactivity. All rates, substrates, and products are established for different substrates, *v*, which can correspond to correct (or cognate, [*c*]) or incorrect (or near-cognate, [*nc*]) substrates.

We initially consider the general diffusion effects on reaction accuracy, based on the scheme in Fig. 1.

Here, diffusion leads to an “encounter complex” (33,34) *E·S*, formed at a net rate *k*_*D*_ and dissociated at a net rate *k*_*-D*_. This is followed by an approximately irreversible reactive step determined by the rate $k_{r}^{v}$. All rates, substrates, and products are established for different substrates, *v*, which can correspond to correct (or cognate, [*c*]) or incorrect (or near-cognate, [*nc*]) substrates (7,35). Under steady-state conditions, in which the significant timescales are long enough to allow equilibration of intermediate reaction states (and with constant reactant concentrations throughout in a continuum limit), the result for product catalysis rate (or flux) is:

(1) $$J^{v}=\frac{d{\left[P\right]}^{v}}{dt}=\frac{k_{D}^{v}k_{r}^{v}\left[E\right]{\left[S\right]}^{v}}{k_{-D}^{v}+k_{r}^{v}}$$

Here, [E] and ${\left[S\right]}^{v}$ are the (constant) concentrations of free enzyme and substrate, respectively (30,36). Minimally, chemical discrimination of one substrate over others requires that $k_{r}^{c}>k_{r}^{nc}$. From Eq. 1, the two kinetic extremes regarding diffusion can be established. The reaction will be chemically limited when $k_{-D}^{v}\gg k_{r}^{v}$, leading to an overall rate of $J_{CL}^{v}=\left(k_{D}^{v}k_{r}^{v}/k_{-D}^{v}\right)\left[E\right]{\left[S\right]}^{v}$. On the other hand, the reaction will be diffusion limited when $k_{-D}^{v}\ll k_{r}^{v}$, leading to a rate of $J_{DL}^{v}=k_{D}^{v}\left[E\right]{\left[S\right]}^{v}$. For set geometric and medium conditions, it follows that $J_{DL}^{v}>J_{CL}^{v}$. A simple way of quantifying the specificity of a reaction is the ratio of the cognate product rate of formation to those of near-cognate products, termed the accuracy (7,35,37,38). From this definition we can see that the reaction accuracy in the presence of various near-cognate substrates is:

(2) $$A=\frac{J^{c}}{\sum_{i}J_{i}^{nc}}={\left\{\underset{i}{\sum }\left(\frac{k_{D,\;i}^{nc}}{k_{D}^{c}}\right)\left[\frac{1+\frac{k_{-D}^{c}}{k_{r}^{c}}}{1+\frac{k_{-D,\;i}^{nc}}{k_{r,i}^{nc}}}\right]\frac{{\left[S\right]}_{i}^{nc}}{{\left[S\right]}^{c}}\right\}}^{-1}$$

The reactive discrimination for a specific substrate is implicit in the factor $k_{-D}/k_{r}$. Consideration of other intermediate, reversible chemical steps leads to additional factors that can be absorbed into the single reactive discrimination factor, provided there are no restart pathways from intermediate steps (37) (see section “Intermediate stages” in the Supporting material). Restart pathways (such as those observed in kinetic proofreading) lead to factors multiplying the entire flux (see section “Protein synthesis” in the Supporting material). Generally, the factors $k_{D}$ and $k_{-D}/k_{r}$ and how they compare between substrates will determine the impact of spatial diffusion on accuracy. A global review of enzyme efficiencies (39) has shown that, on average, cognate reactions *in vitro* are chemically limited ($k_{-D}^{c}\gg k_{r}^{c}$). However, in the crowded cellular environment, viscosities for average-sized proteins can be thousands of times larger than those in aqueous conditions (40), reducing $k_{-D}^{c}$ drastically. This suggests that the average enzyme *in vivo* can operate in a mixed kinetic regime, where diffusion and reactive rates are comparable.

### Spherical geometry

**Figure 2:**
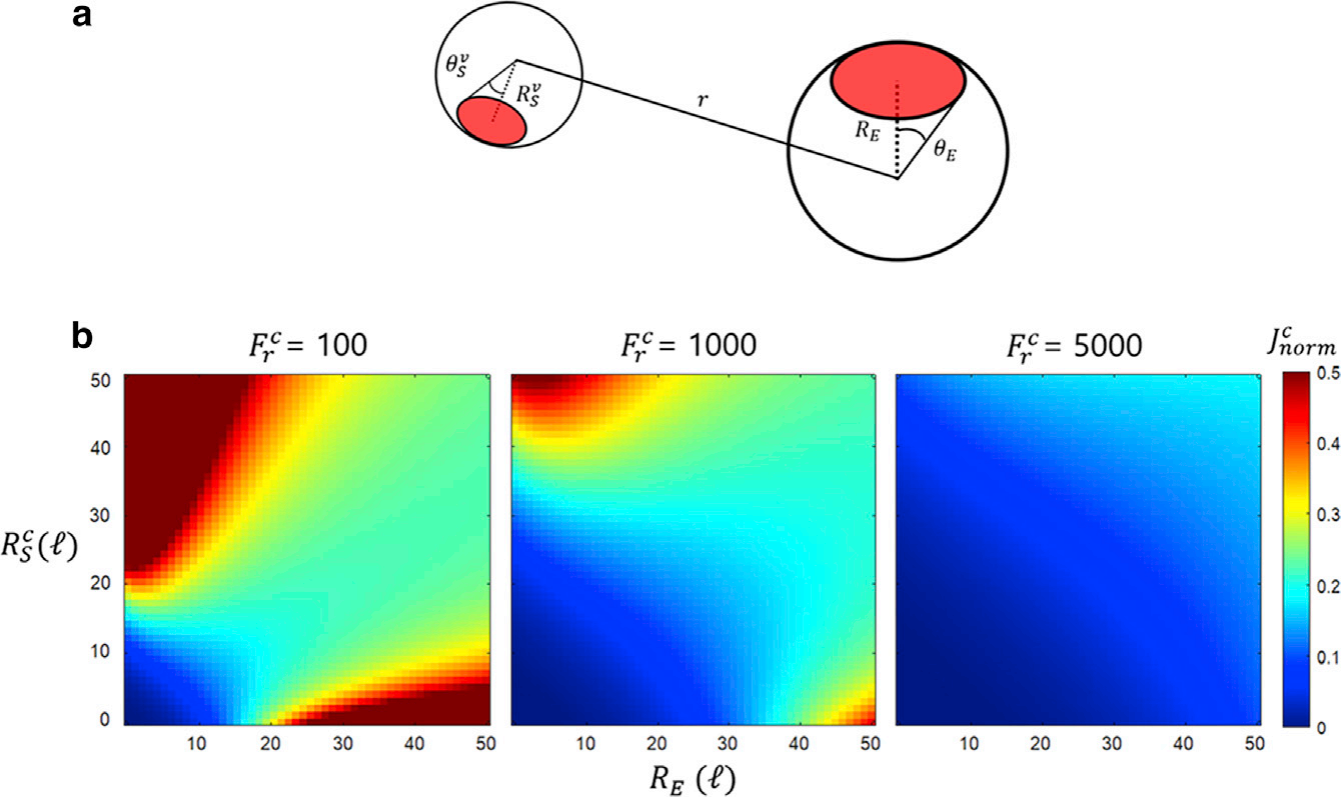
Basic geometric model and rate dependence on reactant geometry. (*A*) The model considers the enzyme and cognate/near-cognate substrates as spheres with uneven reactivity, with radii $R_{E}$ and $R_{S}^{v}$. The reactive areas (red) are spherical caps determined by the polar angles $\theta_{E}$ and $\theta_{S}^{v}$. (*B*) Heatmaps of the normalized cognate product formation rate $J_{norm}^{c}=J^{c}/\left(2k_{B}T\left[E\right]{\left[S\right]}^{c}/3\eta \right)$ as a function of the cognate substrate ($R_{S}^{c}$) and enzyme ($R_{E}$) radii. The radii are expressed in length $\ell ={\left[k_{B}T/2\pi \eta k_{0}\right]}^{1/3}$ for an arbitrary rate $k_{0}$. Parameters were set to $\theta_{E}=10{}^{\circ},\;\theta_{S}^{c}=90{}^{\circ}$. The maps are presented for various reactive discrimination factors $F_{r}^{c}=k_{0}/k_{r}^{c}$, shifting from diffusion-limited reactions (left) to chemically limited reactions (right).

The simplest geometry to describe diffusive association is that of spherical reactants. To the initial model, we integrated the quasi-chemical approximation of Šolc and Stockmayer (23). This scheme is an approximation that accounts for the considerable rotational effects (2) in enzymes and substrates with reactive regions of the surface that are chemically able to interact with each other. In this scheme, enzyme and substrates are modeled as spheres (radii $R_{E}$, $R_{S}^{v}$) with $\varphi_{E}$ and $\varphi_{S}^{v}$ as the reactive surface fractions of the enzyme and substrates, respectively, determined by the polar angles $\theta_{E}$ and $\theta_{S}^{v}$ (Fig 2 *A*). The effect of this geometry on the net diffusion rates is determined by the relations $k_{D}^{v}=\varphi_{S}^{v}\varphi_{E}k_{S}^{v}/\Omega^{v}$ and $k_{-D}^{v}=k_{-S}^{v}/\Omega^{v}$. Here, $\Omega^{v}\left(\leq 1\right)$ are non-trivial factors that measure the likelihood of reactants in contact reorienting into a productive configuration (Eq. S10, section “Omega” in the Supporting material). The factors are therefore maximal ($\Omega^{v}=1$) for uniformly reactive reactants ($\theta_{E}=\theta_{S}^{v}=180{}^{\circ}$). The factors account for the reactive asymmetry and are functions of the ratios between radii ($f^{v}=R_{S}^{v}/R_{E}$) and of the corresponding angles (see section “Spherical geometry” in the Supporting material). The rates $k_{S}^{v}=4\pi \left(D_{S}^{v}+D_{E}\right)\left(R_{S}^{v}+R_{E}\right)$ and $k_{-S}^{v}=3\left(D_{S}^{v}+D_{E}\right)/{\left(R_{S}^{v}+R_{E}\right)}^{2},$ are the diffusive association and dissociation rates for uniformly reactive spheres (41).

The net rates can be interpreted as a decreased association constant ($\varphi_{S}^{v}\varphi_{E}k_{S}^{v}/\Omega^{v}$) and an increased dissociation constant ($k_{-S}^{v}/\Omega^{v}$) stemming from the reduced effective reactive cross section. For uniform reactants, these relations also apply when intermolecular forces are included, only $R=R_{S}^{v}+R_{E}$ is replaced by $1/\overset{\infty }{\underset{R}{\int }}r^{-2}e^{U\left(r\right)/k_{B}T}dr$, where U(r) is the intermolecular potential between enzyme and substrate at a distance *r* from each other (1). In the following calculations, we will assume the interactive and hydrodynamic radii involved in the reaction are similar. This type of consideration points to a few subtleties that are important to consider with respect to the relative scale of different physical forces. For example, it is important to note that the effective radius where Coulomb forces dominate over diffusional forces will increase according to r∝R, where *r* is the distance between the enzyme and substrate. Thus, the effective radius of the substrate or enzyme used in experiments or detailed calculations is likely the measured radius multiplied by a constant factor.

## Results and discussion

### Product flux

Using the relationships for spherical geometries, the resulting expression for the steady-state product formation rate is (see section “Spherical geometry” in the Supporting material):

(3) $$J^{v}=\frac{\varphi_{S}^{v}\varphi_{E}\;\left[2k_{B}T{\left(R_{S}^{v}+R_{E}\right)}^{2}/3\eta R_{S}^{v}R_{E}\right]\left[E\right]{\left[S\right]}^{v}}{\Omega^{v}+\frac{k_{B}T}{2\pi \eta \left(R_{S}^{v}+R_{E}\right)R_{S}^{v}R_{E}k_{r}^{v}}}$$

In this relation, η is the solvent viscosity. Employing this equation, product rates and accuracies can be calculated for different geometric configurations. Fig. 2 *B* shows heatmaps of the normalized cognate product flux, $J^{c}/\left(2k_{B}T\left[E\right]{\left[S\right]}^{c}/3\eta \right)$, as a function of the substrate ($R_{S}^{c}$) and enzyme ($R_{E}$) radii, for fixed reactive areas ($\theta_{E}=10{}^{\circ},\;\theta_{S}^{c}=90{}^{\circ}$) and various reactive discrimination factors $F_{r}^{c}=k_{0}/k_{r}^{c}$, where $k_{0}$ is an arbitrary rate. The radii are expressed in units $\ell ={\left[k_{B}T/2\pi \eta k_{0}\right]}^{1/3}$.

For smaller $F_{r}^{c}$, the reaction is diffusion limited, a condition dictated by the relation $k_{r}^{c}\gg k_{-S}^{c}/\Omega^{c}$, equivalent to $\Omega^{c}R_{S}^{c}R_{E}\left(R_{S}^{c}+R_{E}\right)\gg k_{B}T/2\pi \eta k_{r}^{c}$. The left panel in Fig. 2 *B* is in this kinetic regime, where the rate is approximated by $J_{DL}^{c}=2k_{B}T{\left(R_{S}^{c}+R_{E}\right)}^{2}\varphi_{S}^{c}\varphi_{E}\left[E\right]{\left[S\right]}^{c}/3\eta \Omega^{c}R_{S}^{c}R_{E}$. This rate is fully dictated by diffusive and geometric elements and favors differences in reactant size and larger reactive surface fractions (in this case favoring larger $R_{S}^{c}$ due to its larger reactive surface; see Fig. 2 *B* left panel). This effect is intuitive since it presents an ideal situation for the reactants to diffuse toward each other, namely one small reactant rapidly diffusing toward a larger, slower target. Alternatively, when $\Omega^{c}R_{S}^{c}R_{E}\left(R_{S}^{c}+R_{E}\right)\ll k_{B}T/2\pi \eta k_{r}^{c}$, the reaction is chemically limited, corresponding to the right panel of Fig. 2 *B*. The flux in this case approaches $\;J_{CL}^{c}=4\pi \varphi_{S}^{c}\varphi_{E}{\left(R_{S}^{c}+R_{E}\right)}^{3}k_{r}^{c}\left[E\right]{\left[S\right]}^{c}/3$, which is maximal for larger surface fractions and equal radii (Fig. 2 *B* right panel, upper right corner). These conditions minimize the probability of the reactants dissipating before the chemical reaction can take place, as similarly sized particles will drift apart more slowly than those with large radii differences. Notably, for a specific geometric landscape (with fixed medium conditions η and $k_{B}T$), $J_{DL}^{c}>J_{CL}^{c}$, as can be observed in Fig. 2 *B*.

### Accuracy for different substrate geometries

If we assume that there is a single near-cognate substrate competing with the cognate one, we can compute the accuracy as $A=J^{c}/J^{nc}$ from Eq. 3:

(4) $$A=\left[\frac{\Omega^{nc}+\frac{k_{B}T}{2\pi \eta R_{S}^{nc}R_{E}\left(R_{S}^{nc}+R_{E}\right)k_{r}^{nc}}}{\Omega^{c}+\frac{k_{B}T}{2\pi \eta R_{S}^{c}R_{E}\left(R_{S}^{c}+R_{E}\right)k_{r}^{c}}}\right]\frac{\varphi_{S}^{c}{\left(R_{S}^{c}+R_{E}\right)}^{2}R_{S}^{nc}{\left[S\right]}^{c}}{\varphi_{S}^{nc}{\left(R_{S}^{nc}+R_{E}\right)}^{2}R_{S}^{c}{\left[S\right]}^{nc}}$$

**Figure 3:**
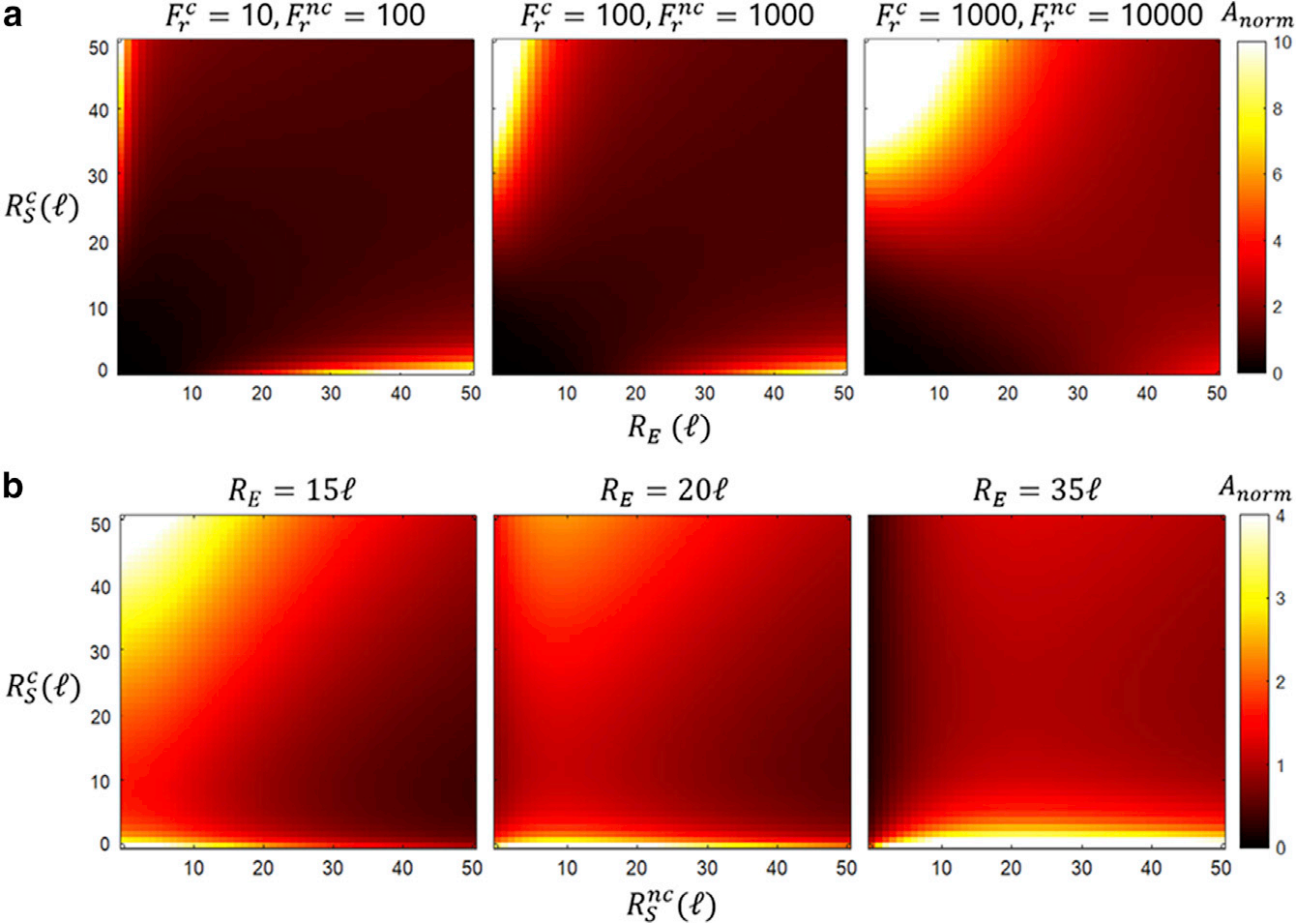
Accuracy for substrates with different geometry. Heatmaps of the normalized accuracy $A_{norm}=\left(J^{c}/J^{nc}\right)/\left({\left[S\right]}^{c}/{\left[S\right]}^{nc}\right)$ for substrates with different geometries are shown as a function of the enzyme ($R_{E})$ or near-cognate substrate ($R_{S}^{nc}$) and the cognate substrate ($R_{S}^{c}$) radii in units $\;\ell ={\left[k_{B}T/2\pi \eta k_{0}\right]}^{1/3}$ for an arbitrary rate $k_{0}.\;$ Parameters were set to $\theta_{E}=10{}^{\circ},\;\theta_{S}^{v}=90{}^{\circ}$. (*A*) Maps are shown for different discrimination factors $F_{r}^{v}=k_{0}/k_{r}^{v}$, shifting from diffusion-limited reactions (left) to chemically limited reactions (right), with constant $R_{S}^{nc}=35\ell$. (*B*) Maps are shown for different enzyme sizes $R_{E}$, with constant factors $F_{r}^{c}=10$ and $F_{r}^{nc}=100$.

Fig. 3 shows heatmaps of the normalized reaction accuracy $A/({\left[S\right]}^{c}/{\left[S\right]}^{nc}$) as a function of the cognate ($R_{S}^{c}$) and enzyme ($R_{E}$) or near-cognate ($R_{S}^{nc}$) radii and for different values of the discrimination factors $F_{r}^{v}$ and enzyme radius $R_{E}$. For these maps, we again use the length unit $\ell ={\left[k_{B}T/2\pi \eta k_{0}\right]}^{1/3}$ (for arbitrary $k_{0}$) and set parameters to $\theta_{E}=10{}^{\circ}$ and $\;\theta_{S}^{v}=90{}^{\circ}$. In Fig. 3 *A* accuracy is mapped as a function of $R_{S}^{c}$ and $R_{E}$ and the values $F_{r}^{v}=k_{0}/k_{r}^{v}$ are increased while maintaining $R_{S}^{nc}=35\ell$ and $F_{r}^{nc}/F_{r}^{c}=k_{r}^{c}/k_{r}^{nc}=10$. The values of $F_{r}^{nc}/F_{r}^{c}$ in biological enzymes are substantially varied, ranging from ∼10 to 10^6^ (42). Here (and throughout the study) we have selected factors within this range that are adequate for illustrative purposes in the geometric landscape observed. From these maps we can make several observations regarding accuracy.

For given enzyme and substrate sizes, the accuracy will decrease with increasing enzyme reactive area (Fig. S3 *A*, increasing $\theta_{E}$). This is straightforward since the enzyme reactive area increases for both correct and incorrect substrates, and hence binding and catalysis become less selective. When the reactions are diffusion limited ($\Omega^{v}R_{S}^{v}R_{E}\left(R_{S}^{v}+R_{E}\right)\gg k_{B}T/2\pi \eta k_{r}^{v}$), the accuracy behaves as $A^{DL}={\left[S\right]}^{c}R_{S}^{nc}{\left(R_{S}^{c}+R_{E}\right)}^{2}\varphi_{S}^{c}\Omega^{nc}/{\left[S\right]}^{nc}R_{S}^{c}{\left(R_{S}^{nc}+R_{E}\right)}^{2}\varphi_{S}^{nc}\Omega^{c}$, a result solely dependent on reactant geometry. The Fig. 3 *A* left panel is inside this diffusion-limited regime, where maximum accuracy is achieved for large reactant size differences (upper left and lower right corners; see section “Accuracy for different geometries” in the Supporting material for specific values). In the case of chemically limited reactions ($\Omega^{v}R_{S}^{v}R_{E}\left(R_{S}^{v}+R_{E}\right)\ll k_{B}T/2\pi \eta k_{r}^{v}$; Fig. 3 *A* right panel), the accuracy approaches the value $A^{CL}={\left[S\right]}^{c}k_{r}^{c}\varphi_{S}^{c}{\left(R_{E}+R_{S}^{c}\right)}^{3}/{\left[S\right]}^{nc}k_{r}^{nc}\varphi_{S}^{nc}{\left(R_{E}+R_{S}^{nc}\right)}^{3}$. In this regime, large reactant size differences still increase accuracy, but with larger cognate substrate sizes relative to the enzyme yielding the maximum (Fig. 3 *A* right panel, upper left corner; see section “Accuracy for different geometries” in the Supporting material for specific values). This shift stems from the increasing contribution to the accuracy of cognate size as reactions become chemically limited. For a specific region of the geometric landscape discussed (with fixed η and $k_{B}T$), the relation $A^{DL}<A^{CL}$ holds if $k_{r}^{nc}/k_{-D}^{nc}<k_{r}^{c}/k_{-D}^{c}$, implying substrate selectivity after binding. These patterns for accuracy are preserved for different near-cognate substrate sizes (Fig. S4). From these results, it is notable that accuracy can display strong dependence on geometric properties even for chemically limited reactions.

In Fig. 3 *B*, accuracy is mapped as a function of $R_{S}^{c}$ and $R_{S}^{nc}$, while $R_{E}$ is varied, maintaining $F_{r}^{c}=10$ and $F_{r}^{nc}=100$, and showing how accuracy is affected by near-cognate geometry. Importantly, for a given enzyme and cognate substrate size, the accuracy is maximal for a specific near-cognate substrate geometry ($R_{S}^{nc}$, $\theta_{S}^{nc}$) dependent on enzyme geometry ($R_{E}$, $\theta_{E}$) and substrate reactivity $k_{r}^{nc}$, as determined by Eq. 4. In the simplest case, for uniformly reactive spheres ($\theta_{S}^{nc}=\theta_{E}=180{}^{\circ},\;\Omega^{nc}=1)$, the enzyme best discriminates substrates of radii $R_{S}^{nc,\;opt}=R_{S}^{nc*}={\left[R_{E}^{2}-\left(3k_{B}T/2\pi \eta R_{E}k_{r}^{nc}\right)\right]}^{1/2}$, or $R_{S}^{nc,\;opt}=0$ (when $R_{S}^{nc*}$ is not real), as these values produce a minimal $J^{nc}$. In the more realistic case where the reactants are not uniformly reactive, the substrate geometry-reactivity combinations that will be best discriminated will be non-trivial (see section “Optimal substrate discrimination” in the Supporting material). This behavior can be observed as either accuracy decreasing with increasing $R_{S}^{nc}$ ($R_{S}^{nc,opt}=0$; Fig. 3 *B* left panel) or as a quick rise and slow decrease in accuracy with increasing $R_{S}^{nc}$ ($R_{S}^{nc,opt}>0$; Fig. 3 *B* center and right panels).

### Accuracy for similar substrate geometries

A particularly significant case of this model is that of cognate and near-cognate substrates of similar geometries ($R_{S}^{c}=R_{S}^{nc}=R_{S}$, $\theta_{S}^{c}=\theta_{S}^{nc}=\theta_{S}$), as in polymerases or the ribosome. In this case, the accuracy (for a single near-cognate species) is simplified to:

(5) $$A=\left[\frac{\frac{\Omega 2\pi \eta R_{S}R_{E}\left(R_{S}+R_{E}\right)}{k_{B}T}+\frac{1}{k_{r}^{nc}}}{\frac{\Omega 2\pi \eta R_{S}R_{E}\left(R_{S}+R_{E}\right)}{k_{B}T}+\frac{1}{k_{r}^{c}}}\right]\frac{{\left[S\right]}^{c}}{{\left[S\right]}^{nc}}$$

**Figure 4:**
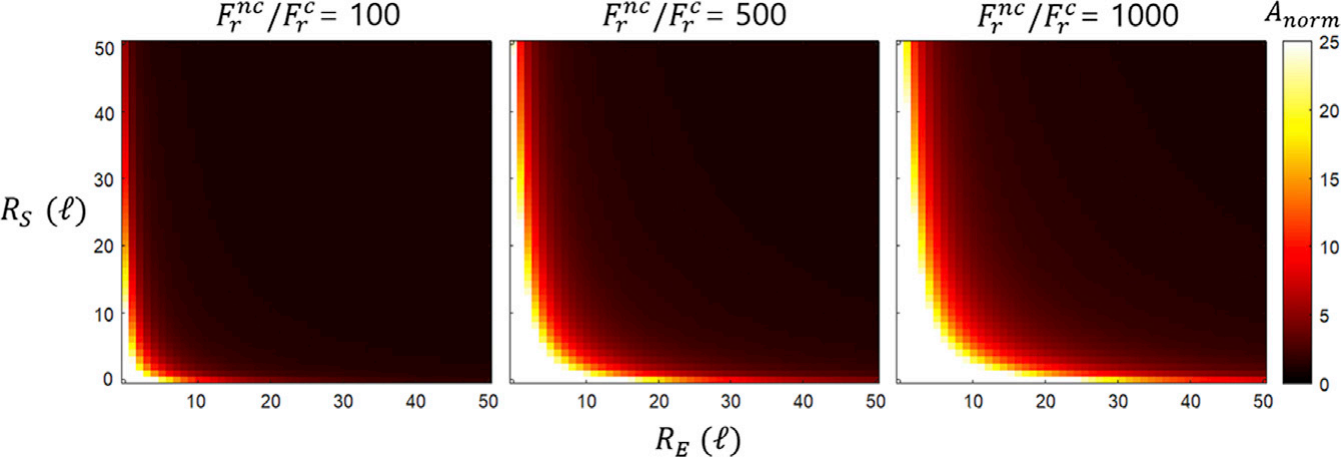
Accuracy for substrates with the same geometry. Heatmaps of the normalized accuracy $A_{norm}=\left(J^{c}/J^{nc}\right)/\left({\left[S\right]}^{c}/{\left[S\right]}^{nc}\right)$ for substrates with equal geometries are shown as a function of the enzyme ($R_{E}$) and substrate ($R_{S}$) radii in units $\ell ={\left[k_{B}T/2\pi \eta k_{0}\right]}^{1/3}$. For these maps, the parameters were set to $\theta_{E}=10{}^{\circ},\;\theta_{S}=90{}^{\circ}$. The maps are presented for various reactive discrimination factor ratios $F_{r}^{nc}/F_{r}^{c}=k_{r}^{c}/k_{r}^{nc}$.

Fig. 4 shows heatmaps of the normalized accuracy $A/({\left[S\right]}^{c}/{\left[S\right]}^{nc}$) as a function of the substrate ($R_{S}$) and enzyme ($R_{E}$) radii. Throughout the calculations, parameters were set to $\theta_{S}=90{}^{\circ},\;\theta_{E}=10{}^{\circ}$. For given reactant geometries, the accuracy will decrease with the increasing reactive area (increasing either $\theta_{E}$ or $\theta_{S}$; see Fig. S3 *B*). This is again because this total reactive cross section increases equally for both correct and incorrect substrates, decreasing net selectivity. When the reactions are diffusion limited, $\Omega R_{S}R_{E}\left(R_{S}+R_{E}\right)\gg k_{B}T/2\pi \eta k_{r}^{v}$, the accuracy will be reduced to ${\left[S\right]}^{c}/{\left[S\right]}^{nc}$, approached in Fig. 4, upper right corners of panels. Chemically limited reactions imply that $\Omega R_{S}R_{E}\left(R_{S}+R_{E}\right)\ll k_{B}T/2\pi \eta k_{r}^{v}$, corresponding to Fig. 4, bottom and left edges of panels. At these extrema, the accuracy approaches the value $A^{‡}={\left[S\right]}^{c}k_{r}^{c}/{\left[S\right]}^{nc}k_{r}^{nc}$. This value will of course be the maximum accuracy if there is any chemical discrimination. Notably, at the geometric landscape origin $\left(R_{S},R_{E}\right)=\left(0,0\right)$, accuracy is maximal ($A=A^{‡}$) while cognate flux is zero (Eq. 3). In this system, as reactants diffuse apart faster (the larger $k_{-D}$ is), the dissolution of the encounter complex overcomes the reactive rate, making the reactions more selective and maximizing the accuracy.

### Effects of viscosity

From Eq. 3, the product formation rate decreases with increasing viscosity η. In the case of accuracy for different cognate and near-cognate substrate geometries (Eq. 4), higher viscosity will be detrimental to the accuracy if the condition $k_{r}^{nc}/k_{-D}^{nc}<k_{r}^{c}/k_{-D}^{c}$ is met (see section “Effects of viscosity” in the Supporting material). This states that, for reactions with substrate selectivity following binding, increasing viscosity will decrease accuracy. As previously observed, this condition also implies that (for fixed viscosity and temperature) the diffusion-limited accuracy will be lower than the chemically limited accuracy ($A^{DL}<A^{CL}$). Therefore, for substrates with similar geometry (Eq. 5), higher viscosity will decrease accuracy if there is any chemical discrimination ($k_{r}^{c}>k_{r}^{nc}$).

### Protein synthesis

Bacterial protein synthesis can be employed as an example for the effects of diffusion on biochemical accuracy, dictated by the developed model for substrates with similar geometry. In this approximation, the enzyme is the ribosome, and the substrates are the ternary complexes, composed of elongation factor Tu (EF-Tu), cognate (or near-cognate) aminoacylated tRNA (aatRNA), and GTP. To obtain the system kinetics, we use *in vitro Escherichia coli* translation rates at 37°C that have been compiled from various measurements by Rudorf et al. (43) (Table S1). Using these rates, the accuracy with aqueous viscosity (as in the buffers in which the experiments were performed) is $A_{aq}=727\pm 236$. Setting the approximate geometric parameters (44) $R_{S}=1.5$ nm, $R_{E}=11$ nm, $\theta_{S}=30{}^{\circ},\;\theta_{E}=20{}^{\circ}$ and viscosities for the reactants in the bacterial cytoplasm (40) (section “Protein synthesis” in the Supporting material), the calculated accuracy in the cell is $A_{cell}=309\pm 100$. The calculated cell result is comparable with accuracies from near-cognate common misincorporation rates *in vivo* (45) corresponding to $A\prime_{cell}=361\pm 109$. Interestingly, the ribosome is not an extreme kinetic case regarding diffusion. The net diffusion rate ($k_{-D}$) in the cell is 30 times larger than the near-cognate reaction rate, but only half of the cognate substrate reaction rate (Table S1). This suggests that, even for reactions that are not diffusion controlled, diffusive effects can alter accuracy considerably.

### Optimization of flux and accuracy

**Figure 5:**
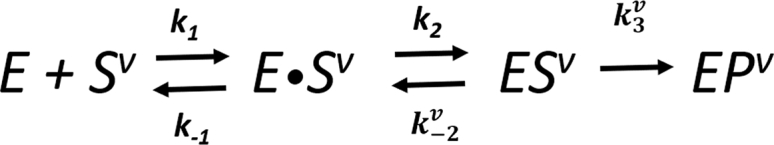
Scheme for optimization of rate and accuracy. Rates, substrates, and products are established for different substrates, *v*, which can correspond to correct (or cognate, [*c*]) or incorrect (or near-cognate, [*nc*]) substrates.

An interesting application of this model is to find combinations of chemical reactivity and geometric arrangement that optimize both flux and specificity. This would increase our understanding of optimal decoding (fast and selective catalysis of the correct substrate) by engineering or evolution of the chemical/diffusive properties of the reactants involved. In the case where there is a single near-cognate substrate, the quantities that must be maximized are the cognate product formation rate $J^{c}$ (Eq. 3) and the accuracy *A* (Eq. 5). Previous work focused on the ribosome has been carried out to find the optimal energy landscape for steady-state rate and accuracy (32), according to the general scheme in Fig. 5.

In this scheme, once more *v* can correspond to cognate or near-cognate substrates and the three stages modeled correspond to binding, recognition, and catalysis. For this general system, the steady-state cognate flux and specificity are negatively correlated (35,46). This property permits the derivation of parameters that optimize the steady-state rate and accuracy simultaneously (i.e., optimal decoding or molecular recognition). The results that we have applied from the study (32) are based on the maximization of the fitness function $F=J^{c}-dJ^{nc}$, where *d* represents the sensitivity of the system to errors. This function was chosen for its simplicity, although the results hold for any biologically reasonable function (see section “Optimization of flux and accuracy” in the Supporting material). As any alteration of the kinetic rates that increases either the rate or the accuracy will decrease the other, the optimum point will be determined by a relationship between the kinetic rates. In this sense, the specific values of the kinetic rates are not important, but rather the relationship between them for the cognate and near-cognate substrates. The key result relates the optimal reactive discrimination factors $F_{r_{2}}^{*}$, namely $F_{r_{2}}^{nc*}={\left(k_{-2}/k_{3}\right)}_{nc}^{*}={\left(k_{3}/k_{-2}\right)}_{c}^{*}\left(1/p^{2}\right)=1/p^{2}F_{r_{2}}^{c*}$, where $p=\frac{k_{-1}}{k_{-1}+k_{2}}$ is the probability of substrate rejection following binding (32). This optimal point can account for diffusive effects through the relation (47) $p={\left[1+\frac{k_{2}}{k_{-b}}\left(1+\frac{k_{b}}{k_{-D}}\right)\right]}^{-1}$, where *k_b_* and *k*_−__*b*_ are the binding and unbinding rates following reactant contact. The influence of spatial diffusion on the optimum is then described quantitatively by:

(6) $$F_{r_{2}}^{nc*}={F_{r_{2}}^{c*}}^{-1}{\left[1+F_{r_{1}}^{-1}\left(1+{F_{s}}^{-1}\right)\right]}^{2}$$

In this expression, the additional discrimination factors equal for all substrates are $F_{r_{1}}=k_{-b}/k_{2}$ (another reactive factor) and $F_{s}=k_{-D}/k_{b}$ (the spatial factor). The rate $k_{-D}$ is (as previously defined) the net rate of the encounter complex diffusing back into the reactants. Eq. 6 therefore determines the ideal relationship between chemical ($F_{r_{1}},\;F_{r_{2}}^{c},\;F_{r_{2}}^{nc}$) and diffusive-geometric ($F_{s}$) discrimination for optimal substrate catalysis flux and accuracy. However, a reaction does not need to have these specific parameters to have optimal flux and accuracy. Less stringently, if ${\left(F_{r_{2}}^{nc}\right)}^{-1}\leq p\leq {\left(F_{r_{2}}^{c}\right)}^{-1}$, the enzyme flux and accuracy will be close to optimal (32), as is the case for the ribosome (see section “Optimization of flux and accuracy” in the Supporting material).

## Conclusion

Enzyme promiscuity is widespread and has considerable mechanistic, evolutionary, and engineering implications (48, 49, 50). Geometric and diffusional limits on transport have direct consequences on the specificity of promiscuous enzymes as the “correct” substrate is selected from the diffusing pool of competing molecules around them. Here we have applied established kinetic expressions (30,36) to explore the diffusive-geometric effects on reaction accuracy in steady-state conditions. These results can be applied to any bimolecular reaction involving discrimination between different reactants. The basic consequence of diffusive effects on accuracy is the inclusion of geometric and solvent properties into the discrimination factors through diffusion rates (Eq. 2). To understand the role of these properties, we applied previously studied non-uniform spherical reactant geometry (24,31) as a first approximation.

Our study shows that, within different kinetic regimes, large variations arise among speed and accuracy optimization on a given geometric landscape. Importantly, even if reactions are chemically limited, accuracy can be strongly dependent on diffusive and geometric factors (see Fig. 3 *A*, right panel). Under diffusion control for a set $R_{S}^{nc}$ different from $R_{S}^{c}$, both the speed and accuracy will increase for large $R_{S}^{c}$ and $R_{E}$ differences (Fig. 2 *B* and Fig. 3 *A* left panels). In contrast, under chemical control, the speed will be optimized for large $R_{S}^{c}$ and $R_{E}$, while the accuracy will favor larger $R_{S}^{c}$ over $R_{E}$ (Fig. 2 *B* and Fig. 3 *A* right panels). Interestingly, for a given enzyme geometry and substrate reactivity ($k_{r}^{nc}$), the enzyme will best discriminate a specific substrate geometry ($R_{S}^{nc}$, $\theta_{S}^{nc}$), as determined by Eq. 4 (see section "Accuracy for different substrate geometries").

For similar substrate geometries, the speed and accuracy are simply optimized in opposite regions of the geometric parameter ($R_{E}$, $R_{S}$) landscape (Fig. 2 *B* and Fig. 4). This speed-accuracy tradeoff falls in line with previous studies based on enzyme kinetic frameworks (5,21,51, 52, 53, 54). Environmental parameters do not necessarily display these tradeoffs, as growing viscosity will diminish both speed and accuracy in reactions with selectivity following binding (see section “Effects of viscosity” in the Supporting material). We have also demonstrated that, in biochemically relevant environments (such as the crowded bacterial cytoplasm), diffusive effects on accuracy can be considerable even for systems far from kinetic extremes (see section “Protein synthesis” in the Supporting material). Finally, starting from previous studies (32), we derived relations between chemical and diffusive rates that optimize the flux and accuracy (see section “Optimization of flux and accuracy” in the Supporting material).

These results explore the chemical, geometric, and diffusive landscape for a promiscuous bimolecular reaction and establish optima for its speed and accuracy. What freedom does a biological or engineered reaction possess to move around this landscape? Biochemical reactions can generally alter chemical rates of binding, recognition, and catalysis through active site mutations (16). The geometric configuration of these reactions is also malleable through the alteration of reactant surfaces (47,55), although reactant size is contingent on factors outside of speed or accuracy (56). Our results suggest that these modifications must take geometric-diffusive effects into account to control speed, specificity, or the balance between them. Inside the possibilities of evolution and engineering, the presented landscape can be navigated to achieve various decoding objectives.

In addition, these results may improve our understanding of enzyme evolution in diverse cellular and biophysical contexts. For example, recent work has shown that the density of cells systematically changes with cell size in bacteria, going from very dense cells at the small end to relatively less dense cells at the large end of bacteria (57). Since viscosity is changing with cell size, the relative size of the two terms being added in A (Eq. 4) also systematically shifts and adjusts the importance of the chemical terms of accuracy. Our prediction is that there should be a much stronger sensitivity to the chemical binding and catalytic rates in large cells compared with small cells, and this should be observable for enzymes that have evolved only in large or small cell species. This type of prediction opens new bioinformatic analyses to characterize the constraints facing the diversity of enzymes across the tree of life.

## Code availability

Computer code used to generate the data that support the findings of this study is available from the corresponding author upon request.

## Author contributions

J.L.A. designed the research. J.L.A., K.P.A., and C.P.K. performed the research. J.L.A., K.P.A., and C.P.K. wrote the paper.

## Supporting material

Document S1. Supporting methods, Figures S1–S4, and Table S1

Document S2. Article plus supporting material

## Footnotes

Supporting material can be found online at https://doi.org/10.1016/j.bpj.2022.03.005.
