# Supplementary material for: Diffusion control in biochemical specificity
Source: Biophys J. 2022 Mar 9;121(8):1541–8. doi: 10.1016/j.bpj.2022.03.005 (PMC9072584; doi:10.1016/j.bpj.2022.03.005)
Supplement: Document S1. Supporting methods, Figures S1–S4, and Table S1 [file mmc1.pdf]

Biophysical Journal, Volume *121*

Supplemental information

Diffusion control in biochemical specificity

Jose L. Alejo, Christopher P. Kempes, and Katarzyna P. Adamala

Diffusion control in biochemical specificity

Jose L. Alejo, Christopher R. Kempes and Katarzyna P. Adamala

Supplementary information

Intermediate stages

If additional reversible stages leading to enzyme catalysis are present, as shown in this scheme:

$$E+S^v \underset{k_{-D}^v}{\overset{k_D^v}{\rightleftharpoons}} E\bullet S^v \underset{k_{-b}^v}{\overset{k_b^v}{\rightleftharpoons}} ES \underset{k_{-1}^v}{\overset{k_1^v}{\rightleftharpoons}} ES^{v\,1} \bullet\bullet\bullet \underset{k_{-(n-1)}^v}{\overset{k_{n-1}^v}{\rightleftharpoons}} ES^{v\,n\text{-}1} \underset{k_{-n}^v}{\overset{k_n^v}{\rightleftharpoons}} ES^{v\,n} \xrightarrow{k_{cat}^v} EP^v$$

The resulting expression for the steady-state flux increases the reactive discrimination factor according to the expression (1):

$$J^v = \frac{k_D^v[E][S]^v}{1 + \frac{k_{-D}^v}{k_b^v}\left(1 + \frac{k_{-b}^v}{k_1^v}\left(1 + \frac{k_{-1}^v}{k_2^v}\left(1 + \cdots + \frac{k_{-(n-1)}^v}{k_n^v}\left(1 + \frac{k_{-n}^v}{k_{cat}^v}\right)\right)\right)\right)} \quad \textbf{(S1)}$$

Spherical geometry

The 'quasi-chemical' approximation (2) model consists of spherical reactants with reactive spherical caps (**Fig. S1**). This model involves various states ($E^{\pm}\bullet S^{\pm}$) connected by rotational motions, including a productive ($E^{+}\bullet S^{+}$) state in which the reactive patches come into contact, allowing catalysis to proceed.

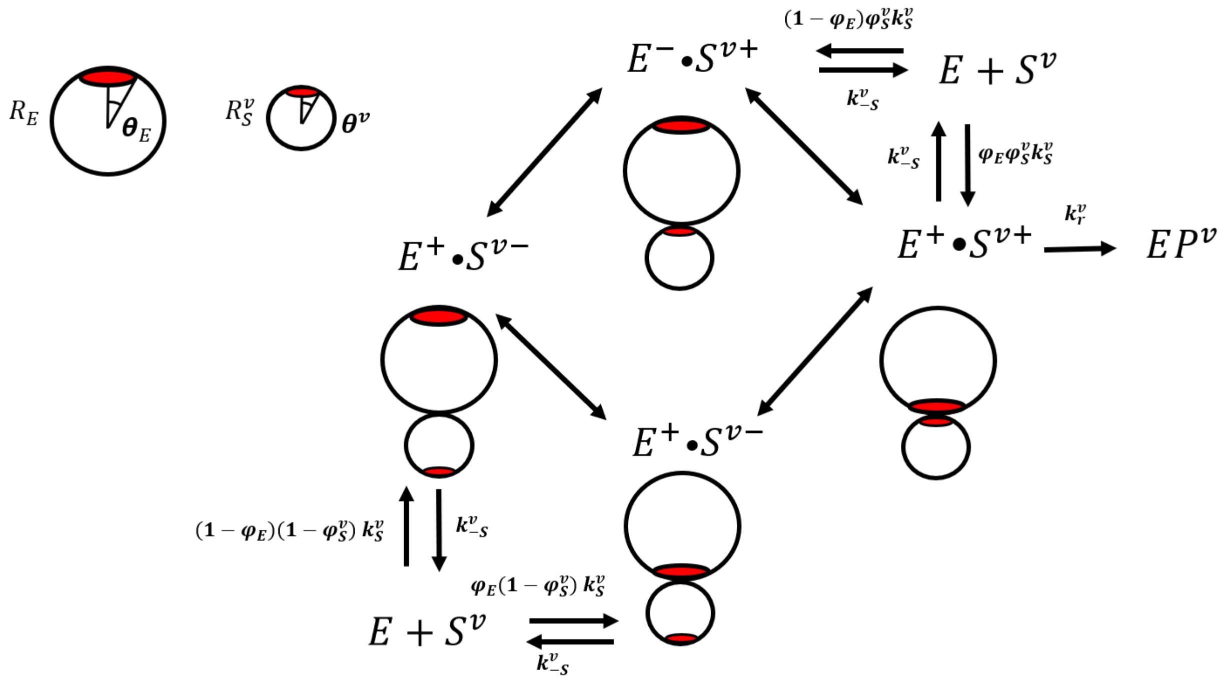

Figure S1. The quasi-chemical model for spherical reactant geometry. Reactant geometries are described by their radii and reactive surface fractions (φ), determined by the polar angles θ. The different states $E^{\pm}\bullet S^{\pm}$ can be formed with modified association constants and interconvert through rotational motions. When the reactive areas contact each other the state $E^{+}\bullet S^{+}$ is formed and the reaction can proceed.

In the model the quantities φ are the reactive surface fractions in the interacting particles, determined by the polar angles θ. Under steady-state assumptions, the main result for the product flux is (Šolc & Stockmayer (2) Equation 1):

$$J = \frac{d[P]}{dt} = k_{eff}[E][S] = \frac{\varphi_E \varphi_S k_S [A][B]}{\Lambda_E \Lambda_S + \psi + \frac{k_{-S}}{k_r}}$$

(S2)

$$\psi = [(1-\Lambda_E)^{-1}(1-\Lambda_S)^{-1} + (1-\Lambda_S)^{-1}(\Lambda_E - \varphi_E)^{-1} + (1-\Lambda_E)^{-1}(\Lambda_S - \varphi_S)^{-1}]^{-1}$$

(S3)

$$\Lambda_{E,S} = \frac{\varphi_{E,S} + k_{-S}\tau_{E,S}}{1 + k_{-S}\tau_{E,S}}$$

(S4)

In their formalism, Šolc and Stockmayer established a net 'chemical' reaction rate k_r, $k_{\pm S}$ are the corresponding rates for spherical reactants and the 'reorientation parameters' $\Lambda_{E,S}$ depend on 'rotational times' (τ_E, τ_S) that are specific to each molecule. An approximation of these parameters has been obtained by comparing k_{eff} in the limit where one of the reactants is uniformly reactive, that is $\varphi_S = 1$. In this limit, $\Lambda_S = 1$ and $\psi = 0$, leading to:

$$J^* = k_{eff}^*[E][S] = \frac{\varphi k_S[E][S]}{\Lambda + \frac{k_{-S}}{k_r}}$$

(S5)

In Berg 1985 (3), this scenario was resolved exactly for spherical molecules. In this model, the reactive fraction of the surface is given by the polar angle θ, namely by the relation $\varphi = (1 - cos\theta)/2$. This gives the result (Berg 1985, Equation 17) for the effective association rate constant, k_a:

$$\frac{k_S}{k_a} = 1 + \left(\frac{1}{\varphi}\right)\left(\frac{D}{\kappa R}\right) - \frac{1}{4\varphi^2}\sum_{j=1}^{\infty}\frac{\left[P_{j-1}(cos\theta) - P_{j+1}(cos\theta)\right]^2}{(2j+1)\left[j - \xi_j K_{j+3/2}(\xi_j)/K_{j+1/2}(\xi_j)\right]}$$

(S6)

In this expression, $P_j(x)$ is the Legendre polynomial of order j and $K_j(x)$ is the modified Bessel function of the second kind of order j. Additionally, $\xi = \sqrt{j(j+1)R^2 D_R/D}$, where D_R is the rotational diffusion coefficient of particle E, $R = R_E + R_S$ is the sum of the hydrodynamic radii, $D = D_E + D_S$ is the sum of the translation diffusion coefficients and κ is the local reactivity per unit area for nonspecific association to the surface of E. By equating k^*_{eff} and k_a, the following relations are obtained:

$$\frac{k_{-S}}{k_r} = \frac{D}{\kappa R}$$

(S7)

$$\Lambda = \varphi - \frac{1}{4\varphi}\sum_{j=1}^{\infty}\frac{\left[P_{j-1}(cos\theta) - P_{j+1}(cos\theta)\right]^2}{(2j+1)\left[j - \xi_j K_{j+3/2}(\xi_j)/K_{j+1/2}(\xi_j)\right]}$$

(S8)

Much simpler approximations of Λ_C exist for diffusion-limited conditions, but this expression is general and (fairly) simple to compute numerically. This expression was used to calculate the reorientation parameters for the enzyme and substrate. This method has been performed in other studies, albeit for the diffusion-limited case (4, 5).

Applying the mentioned results to our situation, we obtain:

$$J^v = \frac{d[P]^v}{dt} = \frac{\varphi_S^v \varphi_E k_S^v [E][S]^v}{\Lambda_E^v \Lambda_S^v + \psi^v + \frac{k_{-S}^v}{k_r^v}} = \frac{\varphi_S^v \varphi_E k_S^v [E][S]^v}{\Omega^v + \frac{k_{-S}^v}{k_r^v}}$$

(S9)

$$\Omega^v = \Lambda_E^v \Lambda_S^v + \psi^v$$

(S10)

As mentioned, an exact solution of the reorientation parameters is given by the expression (3):

$$\Lambda_C = \varphi_C - \frac{1}{4\varphi_C}\sum_{j=1}^{\infty}\frac{\left[P_{j-1}(cos\theta_C) - P_{j+1}(cos\theta_C)\right]^2}{(2j+1)\left[j - \xi_{C,j}K_{j+3/2}(\xi_{C,j})/K_{j+1/2}(\xi_{C,j})\right]}$$

(S11)

In which *C* is *E* or *S*. Using this model, we can broadly explore the effects of diffusion on enzyme accuracy. The basic geometric factors that can affect rate and accuracy are thereby radii (R_E, R_S) and the reactive fractions (φ_E, φ_S) of the molecules. To quantify these effects, the diffusion factors corresponding to spherical reactants are assigned:

$$k_S = 4\pi DR, \qquad k_{-S} = 3D/R^2, \qquad D_C = k_BT/6\pi\eta R_C, \quad D_{C,R} = k_BT/8\pi\eta R_C^3$$

(S12)

In these relations, *D* is the sum of the substrate and enzyme translational diffusion coefficients, which are given by D_C and *R* is the sum of the enzyme and substrate radii. These relations imply that $\xi_{S,j}^E = \sqrt{j(j+1)R^2D_R/D} = \sqrt{j(j+1)3f^{\pm1}(1+f^{\pm1})/4}$, where $f = R_S/R_E$. Under these conditions, the flux is given by (following **Eq. S9**):

$$J^v = \frac{\varphi_S^v\varphi_E\ [2k_BT(R_S^v + R_E)^2/3\eta R_S^v R_E][E][S]^v}{\Omega^v + \dfrac{k_BT}{2\pi\eta(R_S^v + R_E)R_S^v R_E k_r^v}}$$

(S13)

Omega

As described by **Eqs. S10, S11 and S3**, the reorientation factor omega depends on the fraction R_S/R_E and the angles θ_E, θ_S. **Figure S2** shows heat maps of Ω as a function of R_E and R_S, for various reactant angles. In the case of similar angles, similar radii optimize the likelihood of achieving reactive site contact (**Fig. S2** left panel). In contrast, for a reactant with a larger reactive surface fraction, larger radii of this reactant and smaller radii of the other reactant are favored (**Fig. S2** middle and right panels). This difference in reactant sizes maximizes the probability of the reactive regions of the surfaces coming into contact.

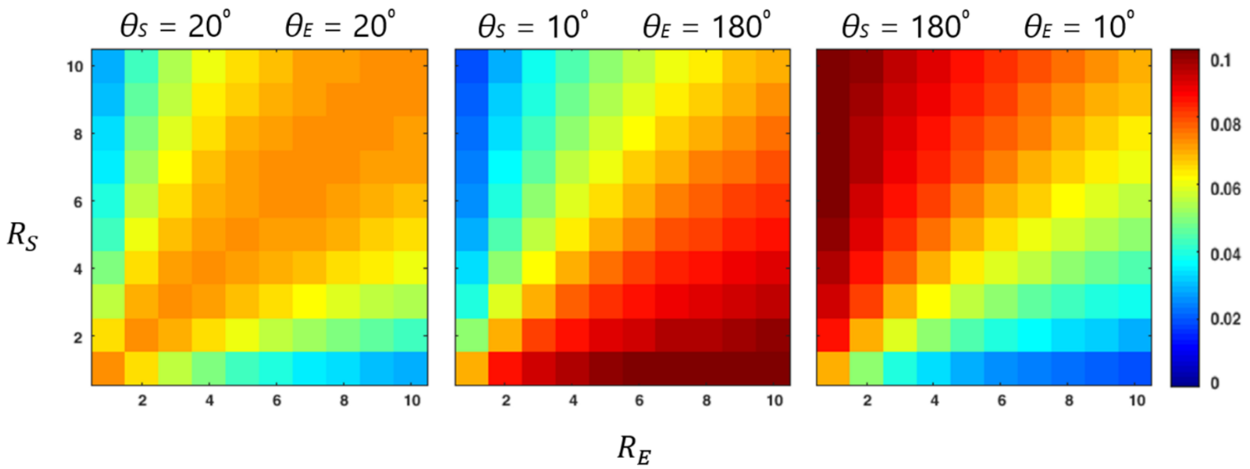

Figure S2. Dependence of the orientation factor omega (Ω) on spherical reactant geometry. Heat maps of the factor omega are shown as a function of the enzyme (R_E) and cognate substrate (R_S) radii in arbitrary length units. Maps are shown for various parameters θ_E and θ_S.

Accuracy for different geometries

As stated in the main text, when reactions are diffusion limited ($\Omega^v R_S^v R_E (R_S^v + R_E) \gg k_B T / 2\pi\eta k_r^v$), the accuracy behaves as $A^{DL} = [S]^c R_S^{nc} (R_S^c + R_E)^2 \varphi_S^c \Omega^{nc} / [S]^{nc} R_S^c (R_S^{nc} + R_E)^2 \varphi_S^{nc} \Omega^c$. For enzyme sizes much larger than the substrates, the accuracy will reach the value $A_1^{DL} = [S]^c R_S^{nc} \varphi_S^c \Omega_0^{nc} / [S]^{nc} R_S^c \varphi_S^{nc} \Omega_0^c$, where the factors $\Omega_0^v = \Omega^v (f^v = R_S^v / R_E = 0)$ are independent of radii. This value is approached in the **Fig. 3a** left panel, lower right corner. On the other hand, substrates much larger than the enzyme lead to the value $A_2^{DL} =$

$[S]^c R_S^c \varphi_S^c \Omega_\infty^{nc} / [S]^{nc} R_S^{nc} \varphi_S^{nc} \Omega_\infty^c$, where $\Omega_\infty^v = \Omega^v(f^v = R_S^v / R_E = \infty)$ are independent of radii. This maximum can be seen in the **Fig. 3a** left panel, upper left corner. In the case of chemically-limited reactions ($\Omega^v R_S^v R_E (R_S^v + R_E) \ll k_B T / 2\pi\eta k_r^v$, **Fig. 3a** right panel), the accuracy behaves as $A^{CL} = [S]^c k_r^c \varphi_S^c (R_E + R_S^c)^3 / [S]^{nc} k_r^{nc} \varphi_S^{nc} (R_E + R_S^{nc})^3$. For enzyme sizes much larger than the substrates, the accuracy will reach the value $A_1^{CL} = [S]^c k_r^c \varphi_S^c / [S]^{nc} k_r^{nc} \varphi_S^{nc}$. This value is approached in the **Fig. 3a** right panel, lower right corner. Alternatively, substrates much larger than the enzyme lead to the value $A_2^{CL} = [S]^c k_r^c \varphi_S^c (R_S^c)^3 / [S]^{nc} k_r^{nc} \varphi_S^{nc} (R_S^{nc})^3$, as can be seen in **Fig. 3a** right panel, upper left corner. Finally, similar large values of the cognate substrate and the enzyme produce another local maximum, $A_3^{CL} = 8[S]^c k_r^c \varphi_S^c / [S]^{nc} k_r^{nc} \varphi_S^{nc}$, approached in the **Fig. 3a** right panel, upper right corner.

Effects of viscosity

The accuracy for different substrate geometries (**Eq. 4**) will decrease with viscosity if $\frac{\partial A}{\partial \eta} < 0$. For an accuracy of the form $A \, \alpha \, \frac{a\eta + b}{c\eta + d}$, with positive coefficients a, b, c, d, the condition $\frac{\partial A}{\partial \eta} < 0$ requires that $da < bc$. The last inequality is equivalent to $k_r^{nc} / k_{-D}^{nc} < k_r^c / k_{-D}^c$ (explicitly, $\Omega^{nc} R_S^{nc} R_E (R_S^{nc} + R_E) / \Omega^c R_S^c R_E (R_S^c + R_E) < k_r^c / k_r^{nc}$). This states that for reactions with substrate selectivity following binding, higher viscosity will decrease accuracy. This directly implies that for substrates with similar geometry (**Eq. 5**), higher viscosity will decrease accuracy if there is any chemical discrimination ($k_r^c > k_r^{nc}$).

Optimal substrate discrimination

To deduce the (near-cognate) substrate geometry that is best discriminated by an enzyme, the general expression for accuracy (**Eq. 4**) can be maximized for varying R_S^{nc}. For local maxima inside the working range of the variable ($R_S^{nc} > 0$), this is directly equivalent to maximizing $1/J^{nc}$ with respect to R_S^{nc}, with the result:

$$(R_S^{nc} + R_E)\left[(2R_S^{nc}R_E + R_E^2)\Omega^{nc} + \frac{\partial\Omega^{nc}}{\partial R_S^{nc}} R_S^{nc} R_E (R_S^{nc} + R_E)\right]$$

$$= 3\left[R_S^{nc} R_E (R_S^{nc} + R_E)\Omega^{nc} + \frac{k_B T}{2\pi\eta k_r^{nc}}\right]$$

(S14)

In the simplest case, for uniformly reactive spheres ($\theta_S^{nc} = \theta_E = 180^{\circ}$, $\Omega^{nc} = 1$), the positive solution is given by:

$$R_S^{nc*} = \left({R_E}^2 - \frac{3k_B T}{2\pi\eta R_E k_r^{nc}}\right)^{1/2}$$

(S15)

Protein synthesis

The ribosome carries out protein synthesis accurately through mRNA decoding by aminoacyl-tRNA (aatRNA). This is accomplished through codon-anticodon recognition, engagement of the codon-anticodon complex by the ribosome and a proofreading step. The 'ternary complex', composed of elongation factor Tu (EF-Tu), aatRNA (either cognate or near-cognate) and GTP (EF-Tu(GTP)-aatRNA) engages the ribosome. Complexes carrying the codon-programmed cognate aatRNA are likely activated for GTP hydrolysis on EF-Tu, while near-cognate complexes are more likely to leave the ribosome before this occurs. Additionally, after GTP hydrolysis the near-cognate complexes are similarly more likely to be released prior to peptide bond formation. In the following scheme, *E* represents the ribosome and S^v represents the cognate (or near-cognate) ternary complex:

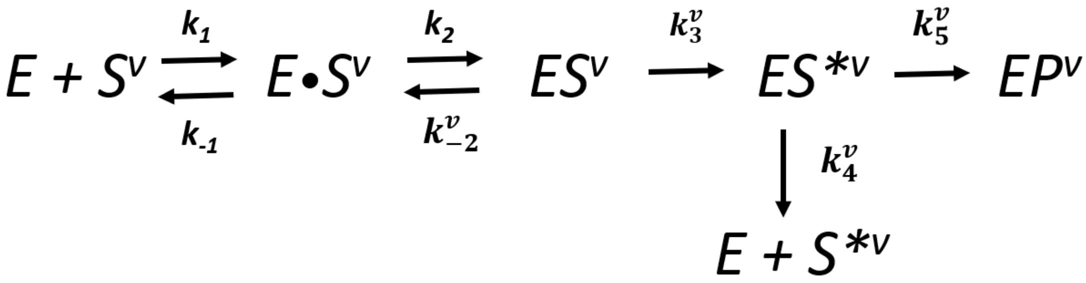

Here, the net rates of diffusive association and binding or diffusive dissociation and unbinding of the two reactants into the encounter ($E \bullet S^v$) complex are compiled into the rates $k_1 = k_D/(1 + k_{-D}/k_b)$ or $k_{-1} = k_{-b}/(1 + k_b/k_{-D})$, respectively (5). Importantly, these rates do not encompass codon recognition, solely the initial binding/unbinding steps of the ternary complex to the ribosome. The rates k_2 and k_{-2}^v determine the codon recognition step and lead to formation of the intermediate denoted as ES^v. From this state, factor GTP hydrolysis occurs with a rate of k_3^v, leading to the formation of intermediate ES^{*v}, corresponding to the ribosome engaged to EF-Tu(GDP)-aatRNA. From this intermediate, S^{*v} can be released at a rate of k_4^v. Once the S^{*v} complex is rejected, reintegration is assumed to be negligible. Finally, the rate k_5^v is the net forward rate of peptidyl transfer leading to the cognate (or near-cognate) product, the peptide chain with the correct (or incorrect) encoded amino acid incorporated (EP^v). In this model, employing the results from the spherical quasi-chemical model, the steady-state product formation rate or product flux is given by:

$$J^v = \frac{k_S \varphi_S \varphi_E [E][S]^v}{\left[\Omega + \frac{k_{-S}}{k_b}\left(1 + \frac{k_{-b}}{k_2}\left(1 + \frac{k_{-2}^v}{k_3^v}\right)\right)\right]\left(1 + \frac{k_4^v}{k_5^v}\right)}$$

(S16)

Where k_S, k_{-S}, φ_S, φ_E and Ω are as defined in the section '**Spherical geometry**'. The accuracy is then given by (for a single near-cognate species):

$$A = \left[\frac{\frac{\Omega k_b}{k_{-D}} + 1 + \frac{k_{-b}}{k_2}\left(1 + \frac{k_{-2}^{nc}}{k_3^{nc}}\right)}{\frac{\Omega k_b}{k_{-D}} + 1 + \frac{k_{-b}}{k_2}\left(1 + \frac{k_{-2}^{c}}{k_3^{c}}\right)}\right] * \left[\frac{1 + \frac{k_4^{nc}}{k_5^{nc}}}{1 + \frac{k_4^{c}}{k_5^{c}}}\right] \frac{[S]^c}{[S]^{nc}} = I * P \frac{[S]^c}{[S]^{nc}}$$

(S17)

This expression for total accuracy is divided into accuracy achieved through the initial (*I*) and proofreading (*P*) selections. We can employ this expression to estimate translation accuracy in cells, and the effects of diffusion on this accuracy. The rates from Rudorf *et al.* (6) in **Table S1** correspond to *in vitro* protein synthesis *E. Coli* kinetic rates at 37°C. The net binding and unbinding rates κ_{on} (k_1) and ω_{off} (k_{-1}) encompass the diffusive and chemical binding steps of these reactions and are given by a specific combination of the diffusive and chemical binding rates. For the buffer conditions that these rates correspond to, we employ the environmental parameter approximation (7) $k_B T/\eta \cong 1.875\text{x}10^9\text{M}^{-1}\text{s}^{-1}$. In the case of the *E. Coli* cell cytoplasm, viscosity for a particle with radius r_p (η_p) in this complex medium is approximated by the phenomenological expression (8):

$$\ln\left(\frac{\eta_p}{\eta_0}\right) = \left(\frac{\varepsilon^2}{R_h^2} + \frac{\varepsilon^2}{r_p^2}\right)^{-a/2}$$

(S18)

In this relation, $R_h = (42 \pm 9)\text{nm}$, $\varepsilon = (0.51 \pm 0.09)\text{nm}$ and $a = 0.53 \pm 0.04$. Using this expression, the diffusion rate k_{-D} in the cell is approximated, and **Eq. S17** is used to

calculate accuracy in the cell. The values and errors of the binding and unbinding rates were calculated using the expressions (5) $k_b = k_{-S}/[(k_S\varphi_S\varphi_E/k_1) - \Omega]$ and $k_{-b} = k_{-1}[1 + (k_b\Omega/k_{-S})]$, yielding $k_b = (2.81 \pm 0.11)\text{x}10^{10}\text{s}^{-1}$ and $k_b = (1119 \pm 105)\text{s}^{-1}$. With these values and those in **Table S1**, we obtained the values and errors of accuracies based on **Equation S17** using a custom MATLAB script.

Rudorf et al. source rate(s) 37°C	***Corresponding rate(s)***	***Units***	***Value***
κ_{on}	k_1	1/μM s	175 ± 25
ω_{off}	k_{-1}	1/s	700 ± 270
ω_{rec}	k_2	1/s	1500 ± 450
ω_{76}	k_{-2}^{nc}	1/s	1100 ± 330
ω_{78}	k_3^{nc}	1/s	7 ± 2
ω_{21}	k_{-2}^{c}	1/s	2 ± 0.6
ω_{23}	k_3^{c}	1/s	1500 ± 450
ω_{90}	k_4^{nc}	1/s	4 ± 0.7
ω_{40}	k_4^{c}	1/s	1
ω_{910}	k_5^{nc}	1/s	0.26 ± 0.04
ω_{45}	k_5^{c}	1/s	200 ± 40

Table S1. The rates from Rudorf *et al*. and the corresponding rates in the model developed in this study. The rate ω_{40} was estimated assuming it is not rate-limiting (6).

Optimization of flux and accuracy

In the study (11), a fitness function is optimized, maximizing both cognate rate and accuracy. The results are based on the maximization of the fitness function $F = J^c - dJ^{nc}$, where d represents the sensitivity of the system to errors. Any number of 'biologically reasonable' functions (for which $\partial F/\partial J^c > 0$ and $\partial F/\partial J^{nc} < 0$) are plausible. Importantly,

the parameter point obtained (**Equation 6**) will be optimal independently of the specific form of F if the condition $(F_{r_2}^{c}/F_{r_2}^{nc}) \lesssim |\partial F/\partial J^{nc}|/|\partial F/\partial J^{c}| \lesssim (F_{r_2}^{nc}/F_{r_2}^{c})$ is met (see (11) for demonstration). This condition implies that so long as J^{nc} and J^{c} are relevant to the fitness F (that is, $|\partial F/\partial J^{nc}|$ and $|\partial F/\partial J^{c}|$ are not different by too many orders of magnitude), the parameter combination in **Equation 6** will be within the optimal range for all F chosen. Though the focus of this optimization was on steady-state quantities, first-passage time and associated errors (splitting probabilities) can yield additional kinetic properties (12, 13). For the ribosome cognate and near-cognate *in vivo* discrimination factors ($F_{r_2}^{nc} \cong 160$, $F_{r_2}^{c} \cong 1/750$, $p = 0.12$, see **Table S1**), are far from the **Eq. 6** optimum ($F_{r_2}^{nc*} = 1/p^2 F_{r_2}^{c} \cong 52080$). Nonetheless, as previously calculated for *in vitro* conditions (11), the ribosome rate and accuracy is nearly optimal. To understand why this is the case, we consider that to optimize decoding the following normalized function must be maximized to 1:

$$M(\Delta_{nc}, \Delta_c) = \frac{1 + e^{(\Delta_{nc} - \Delta_c)/2}}{e^{|\Delta_c - \Delta^*|} + e^{(\Delta_{nc} - \Delta_c)/2}}$$

(S19)

Where $\Delta_v = \ln F_{r_2}^{v}$ and $\Delta^* = -\ln p - (\Delta_{nc} - \Delta_c)/2$ is the optimal near-cognate discrimination factor. This function has been calculated at 98% for *in vitro* conditions (11). For *in vivo* conditions, $\Delta_c = -6.62$, $\Delta_{nc} = 5.06$ (derived from **Table S1**, see **Protein Synthesis**). With these values, **Eq. S19** yields an optimization of ~95%. Hence, for the ribosome the flux and accuracy are optimal across a broad range of parameters. This happens because the function *M* is maximal for a 'band' of values of width $\Delta_{nc} - \Delta_c$ around the optimum $\Delta_c = \Delta^*$ (11), a parameter region equivalent to $(F_{r_2}^{nc})^{-1} \leq p \leq (F_{r_2}^{c})^{-1}$. This is intuitive since the larger this interval is, the more accurate the reaction is, and *p* can potentially grow larger, slowing down the reaction. In this way, larger accuracy permits smaller rates, keeping the

rate and accuracy optimized. While the ribosome parameters are nowhere near the optimum, they fall inside this interval, rendering the reaction's rate and accuracy highly optimized.

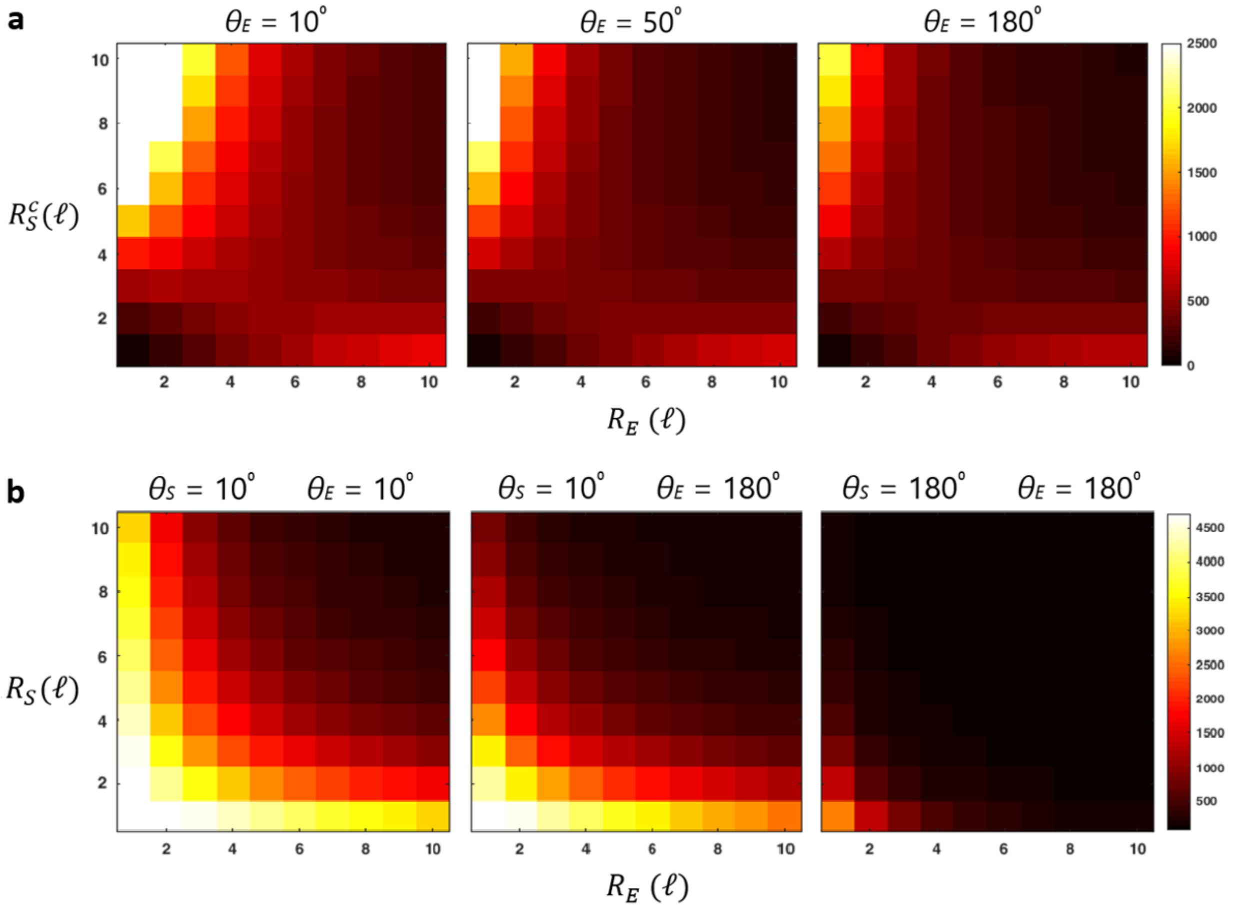

Figure S3. Dependence of accuracy on reactive surface fraction. Radii are displayed in length units $\ell = [k_B T/2\pi\eta k_0]^{1/3}$ for an arbitrary rate k_0 and $F_r^{nc}/F_r^c = 5000$. (**a**) Heat map of the normalized accuracy $A = (J^c/J^{nc})/([S]^c/[S]^{nc})$ for substrates with different geometries for various θ_E. The accuracy is shown as a function of the enzyme (R_E) and cognate substrate (R_S^c) radii. Parameters were set to $\theta_S^v = 10^{\circ}$ and $R_S^{nc} = 3\ell$. (**b**) Heat maps of the normalized accuracy for substrates with equal geometries and different θ_E and θ_S. The accuracy is shown as a function of the enzyme (R_E) and substrate (R_S) radii.

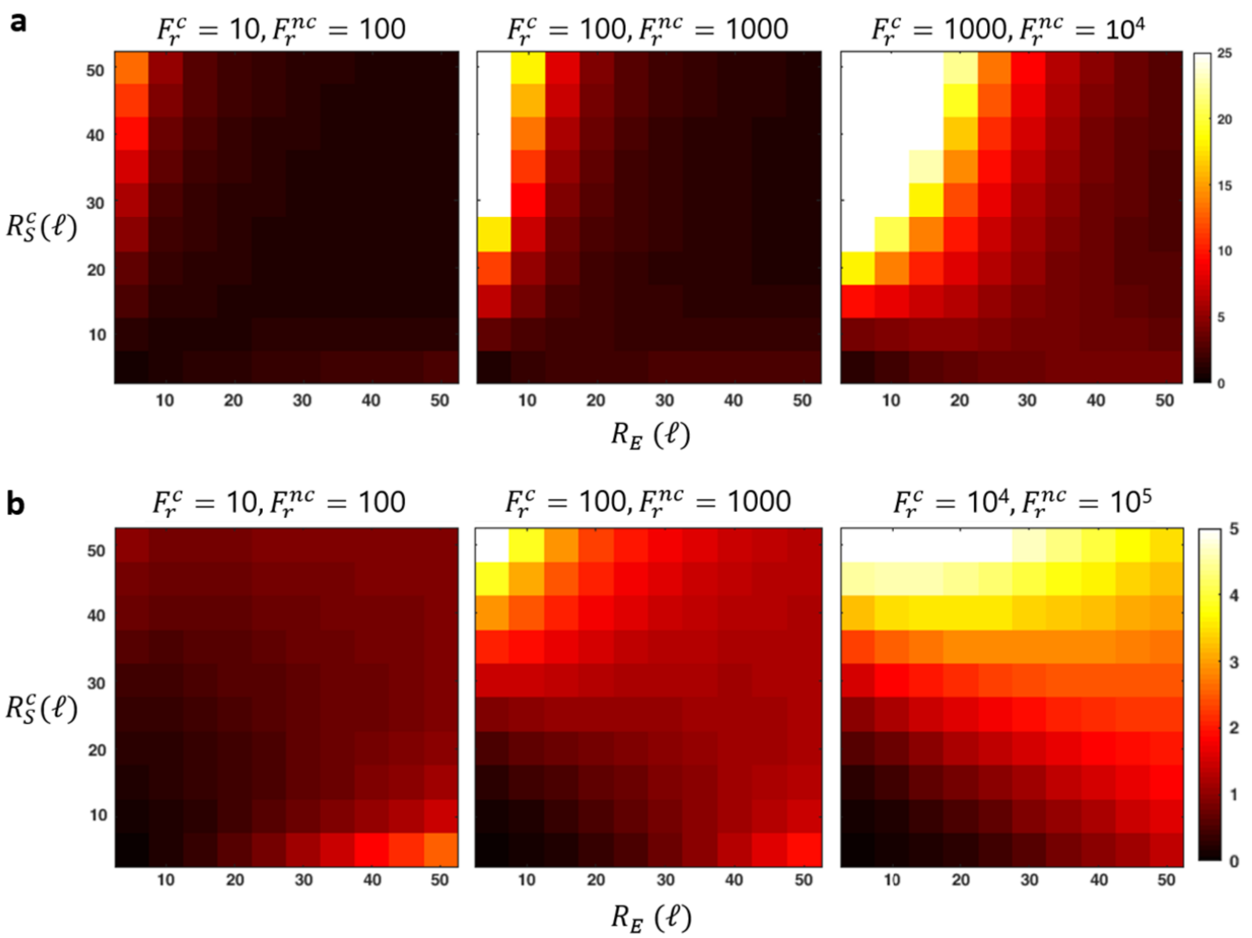

Figure S4. Dependence of accuracy on near-cognate substrate sizes. Heat maps of the normalized accuracy $A = (J^c/J^{nc})/([S]^c/[S]^{nc})$ are shown for substrates with different geometries as a function of the enzyme (R_E) and cognate substrate (R_S^c) radii in length units $\ell = [k_B T/2\pi\eta k_0]^{1/3}$ for an arbitrary rate k_0. Parameters were set to $\theta_E = 10^{\circ}$, $\theta_S^v = 90^{\circ}$. Maps are shown for different discrimination factors $F_r^v = k_0/k_r^v$, shifting from diffusion limited reactions (left panels) to chemically limited reactions (right panels). The maps correspond to near-cognate substrate sizes $R_S^{nc} = 15\ell$ (**a**) and $R_S^{nc} = 60\ell$ (**b**).

REFERENCES

1. Pavlov, M.Y., and M. Ehrenberg. 2018. Substrate-Induced Formation of Ribosomal Decoding Center for Accurate and Rapid Genetic Code Translation. *Annu. Rev. Biophys.* 47:525–548.

2. Šolc, K., and W.H. Stockmayer. 1973. Kinetics of diffusion-controlled reaction

between chemically asymmetric molecules. II. Approximate steady-state solution. *Int. J. Chem. Kinet.* 5:733–752.

3. Berg, O.G. 1985. Orientation Constraints in Diffusion-limited Macromolecular Association: The Role of Surface Diffusion as a Rate-enhancing Mechanism. *Biophys. J.* 47:1–14.
4. Zhou, H. 1993. Brownian dynamics study of the influences of electrostatic interaction and diffusion on protein-protein association kinetics. *Biophys. J.* 64:1711–1726.
5. Nag, A., and A.R. Dinner. 2006. Enhancement of diffusion-controlled reaction rates by surface-induced orientational restriction. *Biophys. J.* 90:896–902.
6. Rudorf, S., M. Thommen, M. V. Rodnina, and R. Lipowsky. 2014. Deducing the Kinetics of Protein Synthesis In Vivo from the Transition Rates Measured In Vitro. *PLoS Comput. Biol.* 10.
7. Berg, O.P. von H. 1985. Diffusion-controlled macromolecular interactions. *Ann Rev Biophys, Biophys Chem*. 14:131–60.
8. Kalwarczyk, T., M. Tabaka, and R. Holyst. 2012. Biologistics-Diffusion coefficients for complete proteome of Escherichia coli. *Bioinformatics*. 28:2971–2978.
9. Johansson, M., J. Zhang, and M. Ehrenberg. 2011. Genetic code translation displays a linear trade-off between efficiency and accuracy of tRNA selection. *Proc Natl Acad Sci*. 2011:1–6.
10. Johansson, M., and M. Lovmar. Rate and accuracy of bacterial protein synthesis revisited ˚ ns Ehrenberg. .
11. Savir, Y., and T. Tlusty. 2013. The ribosome as an optimal decoder: A lesson in molecular recognition. *Cell*. 153:471–479.
12. Banerjee, K., A.B. Kolomeisky, and O.A. Igoshin. 2017. Elucidating interplay of speed and accuracy in biological error correction. *Proc. Natl. Acad. Sci. U. S. A.* 114:5183–5188.

13. van Kampen, N.G. 2007. Stochastic processes in physics and chemistry, 3rd ed. North Holland Publishing Company.
