# Supplementary material for: Diffusion control in biochemical specificity
Source: Biophys J. 2022 Mar 9;121(8):1541–8. doi: 10.1016/j.bpj.2022.03.005 (PMC9072584; doi:10.1016/j.bpj.2022.03.005)
Supplement: Document S2. Article plus supporting — material [file mmc2.pdf]

Biophysical Journal
Article

Biophysical Society

Diffusion control in biochemical specificity

Jose L. Alejo,[1] Christopher P. Kempes,[2] and Katarzyna P. Adamala[1,*]
[1]Department of Genetics, Cell Biology and Development, University of Minnesota, Minneapolis, Minnesota and [2]The Santa Fe Institute, Santa Fe, New Mexico

ABSTRACT Biochemical specificity is critical in enzyme function, evolution, and engineering. Here we employ an established kinetic model to dissect the effects of reactant geometry and diffusion on product formation speed and accuracy in the presence of cognate (correct) and near-cognate (incorrect) substrates. Using this steady-state model for spherical geometries, we find that, for distinct kinetic regimes, the speed and accuracy of the reactions are optimized on different regions of the geometric landscape. From this model we deduce that accuracy can be strongly dependent on reactant geometric properties even for chemically limited reactions. Notably, substrates with a specific geometry and reactivity can be discriminated by the enzyme with higher efficacy than others through purely diffusive effects. For similar cognate and near-cognate substrate geometries (as is the case for polymerases or the ribosome), we observe that speed and accuracy are maximized in opposing regions of the geometric landscape. We also show that, in relevant environments, diffusive effects on accuracy can be substantial even far from extreme kinetic conditions. Finally, we find how reactant chemical discrimination and diffusion can be related to simultaneously optimize steady-state flux and accuracy. These results highlight how diffusion and geometry can be employed to enhance reaction speed and discrimination, and similarly how they impose fundamental restraints on these quantities.

SIGNIFICANCE Biochemical reactions require high specificity in discriminating between similar substrates. Using established chemical kinetic modeling, we explore the role of reactant geometries and diffusion in this specificity. Our results demonstrate that accuracy can be dependent on geometric/diffusive properties even when the underlying reactions are limited by their chemical processes. We show how diffusion can optimally discriminate specific geometry-reactivity combinations of incorrect substrates, as well as the substantial effects of diffusion on specificity in relevant biological scenarios. For similar correct and incorrect substrate geometries (as is the case for polymerases or the ribosome), speed and specificity are maximized in opposing regions of the geometric landscape. Finally, we derive relations between reactivity and diffusion for the simultaneous optimization of speed and accuracy.

INTRODUCTION

It has long been understood that reaction kinetics can be affected by the geometry and diffusion of the participating reactants (1–4). Diffusion is an essential attribute of much of chemistry and biology, imposing fundamental limits on the transport processes of molecular reactants. An aspect of this spatial diffusion that has not been as researched is its effect on enzyme-substrate specificity. Enzymes can engage correct or incorrect substrates and consequently catalyze the formation of distinct products (5,6). High levels of specificity are therefore common in biochemical reactions, and essential in central dogma polymerization processes (7–9).

The accuracy of these enzyme reactions can be quantified by comparing the formation rates of the correct and incorrect products and can be understood in terms of the underlying chemical kinetics (10–14). The basic mechanisms of this substrate discrimination are centered on binding, recognition, and catalysis (15,16), although numerous more complex discrimination mechanisms exist beyond these basic categories, such as catalytic site gating (17–19), kinetic proofreading (20,21), and subsequent quality control (22,23). In an idealized reaction, reactant diffusion entails an additional discrimination mechanism that is implicit in the binding kinetics and independent of the recognition and catalysis stages. These spatial diffusion kinetics are determined by factors such as reactant geometry and reactive sites (24,25), solvent properties (26), molecular crowding (4,27,28), and intermolecular forces (2,29). Successful reactions rates will therefore be limited by the time it takes to achieve the proximity and orientations required for

Submitted May 27, 2021, and accepted for publication March 4, 2022.
*Correspondence: kadamala@umn.edu
Editor: Anatoly B. Kolomeisky.
https://doi.org/10.1016/j.bpj.2022.03.005
© 2022 Biophysical Society.
This is an open access article under the CC BY license (http://creativecommons.org/licenses/by/4.0/).
Check for updates

binding and subsequent catalysis. Under these assumptions, the impact of spatial diffusion on speed and accuracy will depend on how its rates relate to the chemical kinetics that follow.

In this study, we investigate geometric and diffusive effects on the speed and accuracy of promiscuous chemical reactions. Initially, we establish the classical quantitative expressions for rate and accuracy in steady state (10,30). As a general reactant geometric model, we employ the previously characterized framework of asymmetrically reactive spheres (24,31) with varying sizes, reactive sites, and chemical reaction rates. We find that, in specific kinetic regimes, speed and accuracy are optimized in different regions of the geometric landscape (reactant and reactive site sizes). For reactions where the cognate and near-cognate sizes are different, we find the enzyme can discriminate optimally substrates with a specific geometry and reactivity. Alternatively, when the substrates have identical geometries, speed and accuracy are optimized in opposing regions of the geometric landscape. We also apply recently developed expressions (32) to relate chemical and diffusive discrimination and optimize steady-state total flux and accuracy. Finally, we establish that, in biochemically relevant environments, diffusive effects on accuracy (specifically for protein synthesis) can be considerable even far from kinetic extremes. The results derived are an initial approximation to the exploration of wide-ranging relations between chemistry, geometry, diffusion, and accuracy.

METHODS

General expressions

We initially consider the general diffusion effects on reaction accuracy, based on the scheme in Fig. 1.

Here, diffusion leads to an "encounter complex" (33,34) $E \cdot S$, formed at a net rate k_D and dissociated at a net rate k_{-D}. This is followed by an approximately irreversible reactive step determined by the rate k_r^v. All rates, substrates, and products are established for different substrates, v, which can correspond to correct (or cognate, [c]) or incorrect (or near-cognate, [nc]) substrates (7,35). Under steady-state conditions, in which the significant timescales are long enough to allow equilibration of intermediate reaction states (and with constant reactant concentrations throughout in a continuum limit), the result for product catalysis rate (or flux) is:

$$J^v = \frac{d[P]^v}{dt} = \frac{k_D^v k_r^v [E][S]^v}{k_{-D}^v + k_r^v} \tag{1}$$

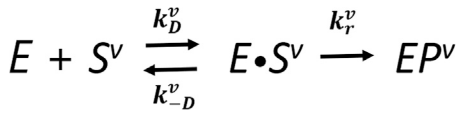

FIGURE 1 Basic scheme of enzyme-substrate diffusion and reactivity. All rates, substrates, and products are established for different substrates, v, which can correspond to correct (or cognate, [c]) or incorrect (or near-cognate, [nc]) substrates.

Here, $[E]$ and $[S]^v$ are the (constant) concentrations of free enzyme and substrate, respectively (30,36). Minimally, chemical discrimination of one substrate over others requires that $k_r^c > k_r^{nc}$. From Eq. 1, the two kinetic extremes regarding diffusion can be established. The reaction will be chemically limited when $k_{-D}^v \gg k_r^v$, leading to an overall rate of $J_{CL}^v = (k_D^v k_r^v / k_{-D}^v)[E][S]^v$. On the other hand, the reaction will be diffusion limited when $k_{-D}^v \ll k_r^v$, leading to a rate of $J_{DL}^v = k_D^v [E][S]^v$. For set geometric and medium conditions, it follows that $J_{DL}^v > J_{CL}^v$. A simple way of quantifying the specificity of a reaction is the ratio of the cognate product rate of formation to those of near-cognate products, termed the accuracy (7,35,37,38). From this definition we can see that the reaction accuracy in the presence of various near-cognate substrates is:

$$A = \frac{J^c}{\sum_i J_i^{nc}} = \left\{ \sum_i \left(\frac{k_{D,\,i}^{nc}}{k_D^c} \right) \left[\frac{1 + \frac{k_{-D}^c}{k_r^c}}{1 + \frac{k_{-D,\,i}^{nc}}{k_{r,i}^{nc}}} \right] \frac{[S]_i^{nc}}{[S]^c} \right\}^{-1} \tag{2}$$

The reactive discrimination for a specific substrate is implicit in the factor k_{-D}/k_r. Consideration of other intermediate, reversible chemical steps leads to additional factors that can be absorbed into the single reactive discrimination factor, provided there are no restart pathways from intermediate steps (37) (see section "Intermediate stages" in the Supporting material). Restart pathways (such as those observed in kinetic proofreading) lead to factors multiplying the entire flux (see section "Protein synthesis" in the Supporting material). Generally, the factors k_D and k_{-D}/k_r and how they compare between substrates will determine the impact of spatial diffusion on accuracy. A global review of enzyme efficiencies (39) has shown that, on average, cognate reactions *in vitro* are chemically limited ($k_{-D}^c \gg k_r^c$). However, in the crowded cellular environment, viscosities for average-sized proteins can be thousands of times larger than those in aqueous conditions (40), reducing k_{-D}^c drastically. This suggests that the average enzyme *in vivo* can operate in a mixed kinetic regime, where diffusion and reactive rates are comparable.

Spherical geometry

The simplest geometry to describe diffusive association is that of spherical reactants. To the initial model, we integrated the quasi-chemical approximation of Šolc and Stockmayer (23). This scheme is an approximation that accounts for the considerable rotational effects (2) in enzymes and substrates with reactive regions of the surface that are chemically able to interact with each other. In this scheme, enzyme and substrates are modeled as spheres (radii R_E, R_S^v) with φ_E and φ_S^v as the reactive surface fractions of the enzyme and substrates, respectively, determined by the polar angles θ_E and θ_S^v (Fig 2 *A*). The effect of this geometry on the net diffusion rates is determined by the relations $k_D^v = \varphi_S^v \varphi_E k_S^v / \Omega^v$ and $k_{-D}^v = k_{-S}^v / \Omega^v$. Here, $\Omega^v (\leq 1)$ are non-trivial factors that measure the likelihood of reactants in contact reorienting into a productive configuration (Eq. S10, section "Omega" in the Supporting material). The factors are therefore maximal ($\Omega^v = 1$) for uniformly reactive reactants ($\theta_E = \theta_S^v = 180°$). The factors account for the reactive asymmetry and are functions of the ratios between radii ($f^v = R_S^v / R_E$) and of the corresponding angles (see section "Spherical geometry" in the Supporting material). The rates $k_S^v = 4\pi(D_S^v + D_E)(R_S^v + R_E)$ and $k_{-S}^v = 3(D_S^v + D_E)/(R_S^v + R_E)^2$, are the diffusive association and dissociation rates for uniformly reactive spheres (41).

The net rates can be interpreted as a decreased association constant ($\varphi_S^v \varphi_E k_S^v / \Omega^v$) and an increased dissociation constant (k_{-S}^v / Ω^v) stemming from the reduced effective reactive cross section. For uniform reactants, these relations also apply when intermolecular forces are included, only $R = R_S^v + R_E$ is replaced by $1 / \int_R^\infty r^{-2} e^{U(r)/k_B T} dr$, where $U(r)$ is the intermolecular potential between enzyme and substrate at a distance r from each

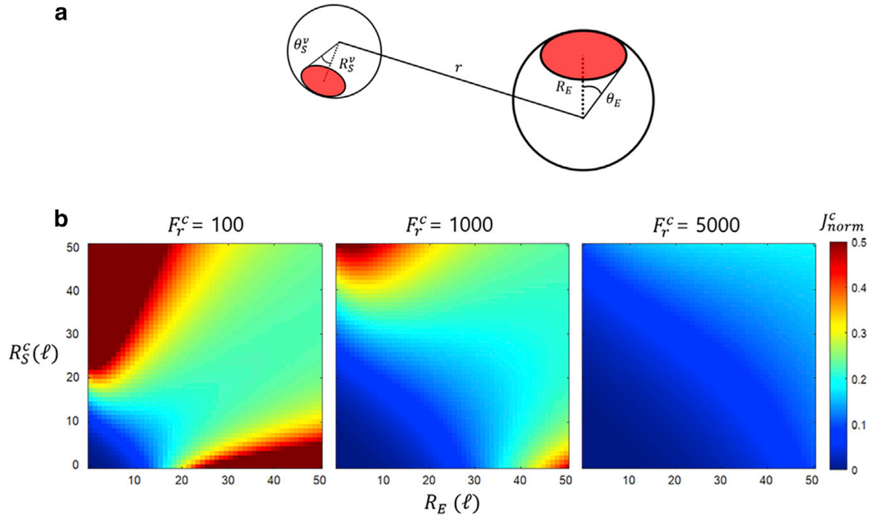

FIGURE 2 Basic geometric model and rate dependence on reactant geometry. (*A*) The model considers the enzyme and cognate/near-cognate substrates as spheres with uneven reactivity, with radii R_E and R_S^v. The reactive areas (red) are spherical caps determined by the polar angles θ_E and θ_S^v. (*B*) Heatmaps of the normalized cognate product formation rate $J_{norm}^c = J^c/(2k_BT[E][S]^c/3\eta)$ as a function of the cognate substrate (R_S^c) and enzyme (R_E) radii. The radii are expressed in length $\ell = [k_BT/2\pi\eta k_0]^{1/3}$ for an arbitrary rate k_0. Parameters were set to $\theta_E = 10°$, $\theta_S^c = 90°$. The maps are presented for various reactive discrimination factors $F_r^c = k_0/k_r^c$, shifting from diffusion-limited reactions (left) to chemically limited reactions (right).

other (1). In the following calculations, we will assume the interactive and hydrodynamic radii involved in the reaction are similar. This type of consideration points to a few subtleties that are important to consider with respect to the relative scale of different physical forces. For example, it is important to note that the effective radius where Coulomb forces dominate over diffusional forces will increase according to $r \propto R$, where r is the distance between the enzyme and substrate. Thus, the effective radius of the substrate or enzyme used in experiments or detailed calculations is likely the measured radius multiplied by a constant factor.

RESULTS AND DISCUSSION

Product flux

Using the relationships for spherical geometries, the resulting expression for the steady-state product formation rate is (see section "Spherical geometry" in the Supporting material):

$$J^v = \frac{\varphi_S^v \varphi_E \left[2k_BT\left(R_S^v + R_E\right)^2 \Big/ 3\eta R_S^v R_E\right][E][S]^v}{\Omega^v + \frac{k_BT}{2\pi\eta\left(R_S^v + R_E\right)R_S^v R_E k_r^v}} \tag{3}$$

In this relation, η is the solvent viscosity. Employing this equation, product rates and accuracies can be calculated for different geometric configurations. Fig. 2 *B* shows heatmaps of the normalized cognate product flux, $J^c/(2k_BT[E][S]^c/3\eta)$, as a function of the substrate (R_S^c) and enzyme (R_E) radii, for fixed reactive areas ($\theta_E = 10°$, $\theta_S^c = 90°$) and various reactive discrimination factors $F_r^c = k_0/k_r^c$, where k_0 is an arbitrary rate. The radii are expressed in units $\ell = [k_BT/2\pi\eta k_0]^{1/3}$.

For smaller F_r^c, the reaction is diffusion limited, a condition dictated by the relation $k_r^c \gg k_{-S}^c/\Omega^c$, equivalent to $\Omega^c R_S^c R_E (R_S^c + R_E) \gg k_BT/2\pi\eta k_r^c$. The left panel in Fig. 2 *B* is in this kinetic regime, where the rate is approximated by $J_{DL}^c = 2k_BT(R_S^c + R_E)^2 \varphi_S^c \varphi_E [E][S]^c / 3\eta\Omega^c R_S^c R_E$. This rate is fully dictated by diffusive and geometric elements and favors differences in reactant size and larger reactive surface fractions (in this case favoring larger R_S^c due to its larger reactive surface; see Fig. 2 *B* left panel). This effect is intuitive since it presents an ideal situation for the reactants to diffuse toward each other, namely one small reactant rapidly diffusing toward a larger, slower target. Alternatively, when $\Omega^c R_S^c R_E (R_S^c + R_E) \ll k_BT/2\pi\eta k_r^c$, the reaction is chemically limited, corresponding to the right panel of Fig. 2 *B*. The flux in this case approaches $J_{CL}^c = 4\pi\varphi_S^c\varphi_E(R_S^c + R_E)^3 k_r^c [E][S]^c/3$, which is maximal for larger surface fractions and equal radii (Fig. 2 *B* right panel, upper right corner). These conditions minimize the probability of the reactants dissipating before the chemical reaction can take place, as similarly sized particles will drift apart more slowly than those with large radii differences. Notably, for a specific geometric landscape (with fixed medium conditions η and k_BT), $J_{DL}^c > J_{CL}^c$, as can be observed in Fig. 2 *B*.

Accuracy for different substrate geometries

If we assume that there is a single near-cognate substrate competing with the cognate one, we can compute the accuracy as $A = J^c/J^{nc}$ from Eq. 3:

$$A = \left[\frac{\Omega^{nc} + \frac{k_BT}{2\pi\eta R_S^{nc} R_E \left(R_S^{nc} + R_E\right) k_r^{nc}}}{\Omega^c + \frac{k_BT}{2\pi\eta R_S^c R_E \left(R_S^c + R_E\right) k_r^c}}\right] \frac{\varphi_S^c \left(R_S^c + R_E\right)^2 R_S^{nc} [S]^c}{\varphi_S^{nc}\left(R_S^{nc} + R_E\right)^2 R_S^c [S]^{nc}} \tag{4}$$

Fig. 3 shows heatmaps of the normalized reaction accuracy $A/([S]^c/[S]^{nc})$ as a function of the cognate (R_S^c) and enzyme (R_E) or near-cognate (R_S^{nc}) radii and for different values of the discrimination factors F_r^v and enzyme radius R_E. For these maps, we again use the length unit $\ell = [k_BT/2\pi\eta k_0]^{1/3}$ (for

arbitrary k_0) and set parameters to $\theta_E = 10°$ and $\theta_S^v = 90°$. In Fig. 3 *A* accuracy is mapped as a function of R_S^c and R_E and the values $F_r^v = k_0/k_r^v$ are increased while maintaining $R_S^{nc} = 35\ell$ and $F_r^{nc}/F_r^c = k_r^c/k_r^{nc} = 10$. The values of F_r^{nc}/F_r^c in biological enzymes are substantially varied, ranging from $\sim$10 to 10^6 (42). Here (and throughout the study) we have selected factors within this range that are adequate for illustrative purposes in the geometric landscape observed. From these maps we can make several observations regarding accuracy.

For given enzyme and substrate sizes, the accuracy will decrease with increasing enzyme reactive area (Fig. S3 *A*, increasing θ_E). This is straightforward since the enzyme reactive area increases for both correct and incorrect substrates, and hence binding and catalysis become less selective. When the reactions are diffusion limited ($\Omega^v R_S^v R_E (R_S^v + R_E) \gg k_B T/2\pi\eta k_r^v$), the accuracy behaves as $A^{DL} = [S]^c R_S^{nc} (R_S^c + R_E)^2 \varphi_S^c \Omega^{nc} / [S]^{nc} R_S^c (R_S^{nc} + R_E)^2 \varphi_S^{nc} \Omega^c$, a result solely dependent on reactant geometry. The Fig. 3 *A* left panel is inside this diffusion-limited regime, where maximum accuracy is achieved for large reactant size differences (upper left and lower right corners; see section "Accuracy for different geometries" in the Supporting material for specific values). In the case of chemically limited reactions ($\Omega^v R_S^v R_E (R_S^v + R_E) \ll k_B T/2\pi\eta k_r^v$; Fig. 3 *A* right panel), the accuracy approaches the value $A^{CL} = [S]^c k_r^c \varphi_S^c (R_E + R_S^c)^3 / [S]^{nc} k_r^{nc} \varphi_S^{nc} (R_E + R_S^{nc})^3$. In this regime, large reactant size differences still increase accuracy, but with larger cognate substrate sizes relative to the enzyme yielding the maximum (Fig. 3 *A* right panel, upper left corner; see section "Accuracy for different geometries" in the Supporting material for specific values). This shift stems from the increasing contribution to the accuracy of cognate size as reactions become chemically limited. For a specific region of the geometric landscape discussed (with fixed η and $k_B T$), the relation $A^{DL} < A^{CL}$ holds if $k_r^{nc}/k_{-D}^{nc} < k_r^c/k_{-D}^c$, implying substrate selectivity after binding. These patterns for accuracy are preserved for different near-cognate substrate sizes (Fig. S4). From these results, it is notable that accuracy can display strong dependence on geometric properties even for chemically limited reactions.

In Fig. 3 *B*, accuracy is mapped as a function of R_S^c and R_S^{nc}, while R_E is varied, maintaining $F_r^c = 10$ and $F_r^{nc} = 100$, and showing how accuracy is affected by near-cognate geometry. Importantly, for a given enzyme and cognate substrate size, the accuracy is maximal for a specific near-cognate substrate geometry (R_S^{nc}, θ_S^{nc}) dependent on enzyme geometry (R_E, θ_E) and substrate reactivity k_r^{nc}, as determined by Eq. 4. In the simplest case, for uniformly reactive spheres ($\theta_S^{nc} = \theta_E = 180°$, $\Omega^{nc} = 1$), the enzyme best discriminates substrates of radii $R_S^{nc,\,opt} = R_S^{nc*} = [R_E^2 - (3k_B T/2\pi\eta R_E k_r^{nc})]^{1/2}$, or $R_S^{nc,\,opt} = 0$ (when R_S^{nc*} is not real), as these values produce a minimal J^{nc}. In the more realistic case where the reactants are not uniformly reactive, the substrate geometry-reactivity combinations that will be best discriminated will be non-trivial (see section "Optimal substrate discrimination" in the Supporting material). This behavior can be observed as either accuracy decreasing with increasing R_S^{nc} ($R_S^{nc,opt} = 0$; Fig. 3 *B* left panel) or as a quick rise and slow decrease in accuracy with increasing R_S^{nc} ($R_S^{nc,opt} > 0$; Fig. 3 *B* center and right panels).

Accuracy for similar substrate geometries

A particularly significant case of this model is that of cognate and near-cognate substrates of similar geometries ($R_S^c = R_S^{nc} = R_S$, $\theta_S^c = \theta_S^{nc} = \theta_S$), as in polymerases or the ribosome. In this case, the accuracy (for a single near-cognate species) is simplified to:

$$A = \left[\frac{\frac{\Omega 2\pi\eta R_S R_E (R_S + R_E)}{k_B T} + \frac{1}{k_r^{nc}}}{\frac{\Omega 2\pi\eta R_S R_E (R_S + R_E)}{k_B T} + \frac{1}{k_r^c}} \right] \frac{[S]^c}{[S]^{nc}} \qquad (5)$$

Fig. 4 shows heatmaps of the normalized accuracy $A/([S]^c/[S]^{nc})$ as a function of the substrate (R_S) and enzyme (R_E) radii. Throughout the calculations, parameters were set to $\theta_S = 90°$, $\theta_E = 10°$. For given reactant geometries, the accuracy will decrease with the increasing reactive area (increasing either θ_E or θ_S; see Fig. S3 *B*). This is again because this total reactive cross section increases equally for both correct and incorrect substrates, decreasing net selectivity. When the reactions are diffusion limited, $\Omega R_S R_E (R_S + R_E) \gg k_B T/2\pi\eta k_r^v$, the accuracy will be reduced to $[S]^c/[S]^{nc}$, approached in Fig. 4, upper right corners of panels. Chemically limited reactions imply that $\Omega R_S R_E (R_S + R_E) \ll k_B T/2\pi\eta k_r^v$, corresponding to Fig. 4, bottom and left edges of panels. At these extrema, the accuracy approaches the value $A^{\ddagger} = [S]^c k_r^c / [S]^{nc} k_r^{nc}$. This value will of course be the maximum accuracy if there is any chemical discrimination. Notably, at the geometric landscape origin $(R_S, R_E) = (0, 0)$, accuracy is maximal ($A = A^{\ddagger}$) while cognate flux is zero (Eq. 3). In this system, as reactants diffuse apart faster (the larger k_{-D} is), the dissolution of the encounter complex overcomes the reactive rate, making the reactions more selective and maximizing the accuracy.

Effects of viscosity

From Eq. 3, the product formation rate decreases with increasing viscosity η. In the case of accuracy for different cognate and near-cognate substrate geometries (Eq. 4), higher viscosity will be detrimental to the accuracy if the condition $k_r^{nc}/k_{-D}^{nc} < k_r^c/k_{-D}^c$ is met (see section "Effects of viscosity" in the Supporting material). This states that, for reactions with substrate selectivity following binding, increasing viscosity will decrease accuracy. As previously

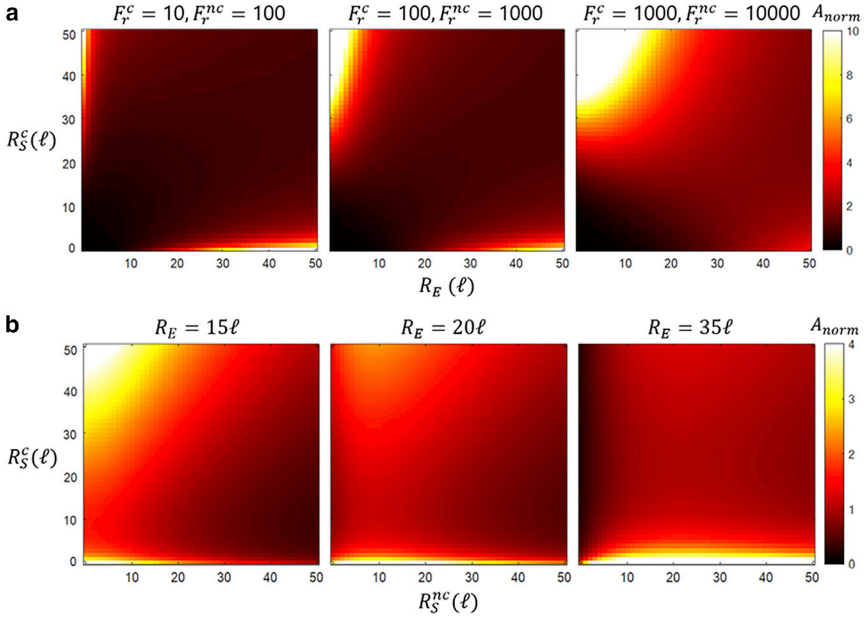

FIGURE 3 Accuracy for substrates with different geometry. Heatmaps of the normalized accuracy $A_{norm} = (J^c/J^{nc})/([S]^c/[S]^{nc})$ for substrates with different geometries are shown as a function of the enzyme (R_E) or near-cognate substrate (R_S^{nc}) and the cognate substrate (R_S^c) radii in units $\ell = [k_B T/2\pi\eta k_0]^{1/3}$ for an arbitrary rate k_0. Parameters were set to $\theta_E = 10°$, $\theta_S^v = 90°$. (*A*) Maps are shown for different discrimination factors $F_r^v = k_0/k_r^v$, shifting from diffusion-limited reactions (left) to chemically limited reactions (right), with constant $R_S^{nc} = 35\ell$. (*B*) Maps are shown for different enzyme sizes R_E, with constant factors $F_r^c = 10$ and $F_r^{nc} = 100$.

observed, this condition also implies that (for fixed viscosity and temperature) the diffusion-limited accuracy will be lower than the chemically limited accuracy ($A^{DL} < A^{CL}$). Therefore, for substrates with similar geometry (Eq. 5), higher viscosity will decrease accuracy if there is any chemical discrimination ($k_r^c > k_r^{nc}$).

Protein synthesis

Bacterial protein synthesis can be employed as an example for the effects of diffusion on biochemical accuracy, dictated by the developed model for substrates with similar geometry. In this approximation, the enzyme is the ribosome, and the substrates are the ternary complexes, composed of elongation factor Tu (EF-Tu), cognate (or near-cognate) aminoacylated tRNA (aatRNA), and GTP. To obtain the system kinetics, we use *in vitro Escherichia coli* translation rates at 37°C that have been compiled from various measurements by Rudorf et al. (43) (Table S1). Using these rates, the accuracy with aqueous viscosity (as in the buffers in which the experiments were performed) is $A_{aq} = 727 \pm 236$. Setting the approximate geometric parameters (44) $R_S = 1.5$ nm, $R_E = 11$ nm, $\theta_S = 30°$, $\theta_E = 20°$ and viscosities for the reactants in the bacterial cytoplasm (40) (section "Protein synthesis" in the Supporting material), the calculated accuracy in the cell is $A_{cell} = 309 \pm 100$. The calculated cell result is comparable with accuracies from near-cognate common misincorporation rates *in vivo* (45) corresponding to $A\prime_{cell} = 361 \pm 109$. Interestingly, the ribosome is not an extreme kinetic case regarding diffusion. The net diffusion rate (k_{-D}) in the cell is 30 times larger than the near-cognate reaction rate, but only half of the cognate substrate reaction rate (Table S1). This suggests that, even for reactions that are not diffusion controlled, diffusive effects can alter accuracy considerably.

Optimization of flux and accuracy

An interesting application of this model is to find combinations of chemical reactivity and geometric arrangement that optimize both flux and specificity. This would increase our understanding of optimal decoding (fast and selective

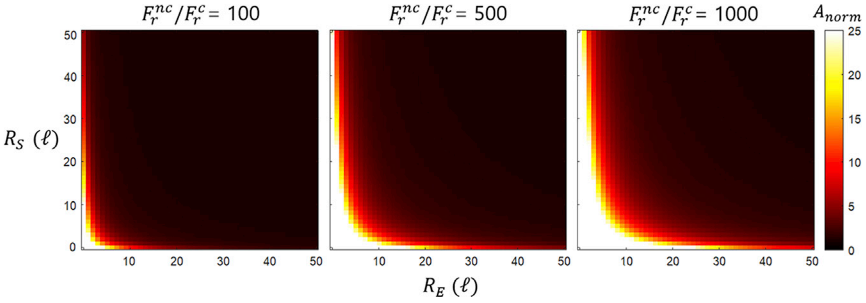

FIGURE 4 Accuracy for substrates with the same geometry. Heatmaps of the normalized accuracy $A_{norm} = (J^c/J^{nc})/([S]^c/[S]^{nc})$ for substrates with equal geometries are shown as a function of the enzyme (R_E) and substrate (R_S) radii in units $\ell = [k_B T/2\pi\eta k_0]^{1/3}$. For these maps, the parameters were set to $\theta_E = 10°$, $\theta_S = 90°$. The maps are presented for various reactive discrimination factor ratios $F_r^{nc}/F_r^c = k_r^c/k_r^{nc}$.

catalysis of the correct substrate) by engineering or evolution of the chemical/diffusive properties of the reactants involved. In the case where there is a single near-cognate substrate, the quantities that must be maximized are the cognate product formation rate J^c (Eq. 3) and the accuracy A (Eq. 5). Previous work focused on the ribosome has been carried out to find the optimal energy landscape for steady-state rate and accuracy (32), according to the general scheme in Fig. 5.

In this scheme, once more v can correspond to cognate or near-cognate substrates and the three stages modeled correspond to binding, recognition, and catalysis. For this general system, the steady-state cognate flux and specificity are negatively correlated (35,46). This property permits the derivation of parameters that optimize the steady-state rate and accuracy simultaneously (i.e., optimal decoding or molecular recognition). The results that we have applied from the study (32) are based on the maximization of the fitness function $F = J^c - dJ^{nc}$, where d represents the sensitivity of the system to errors. This function was chosen for its simplicity, although the results hold for any biologically reasonable function (see section “Optimization of flux and accuracy” in the Supporting material). As any alteration of the kinetic rates that increases either the rate or the accuracy will decrease the other, the optimum point will be determined by a relationship between the kinetic rates. In this sense, the specific values of the kinetic rates are not important, but rather the relationship between them for the cognate and near-cognate substrates. The key result relates the optimal reactive discrimination factors $F^*_{r_2}$, namely $F^{nc*}_{r_2} = (k_{-2}/k_3)^*_{nc} = (k_3/k_{-2})^*_c(1/p^2) = 1/p^2F^{c*}_{r_2}$, where $p = \frac{k_{-1}}{k_{-1}+k_2}$ is the probability of substrate rejection following binding (32). This optimal point can account for diffusive effects through the relation (47) $p = \left[1+\frac{k_2}{k_{-b}}\left(1+\frac{k_b}{k_{-D}}\right)\right]^{-1}$, where k_b and k_{-b} are the binding and unbinding rates following reactant contact. The influence of spatial diffusion on the optimum is then described quantitatively by:

$$F^{nc*}_{r_2} = F^{c*-1}_{r_2}\left[1+F^{-1}_{r_1}\left(1+F_s{}^{-1}\right)\right]^2 \tag{6}$$

In this expression, the additional discrimination factors equal for all substrates are $F_{r_1} = k_{-b}/k_2$ (another reactive factor) and $F_s = k_{-D}/k_b$ (the spatial factor). The rate k_{-D} is (as previously defined) the net rate of the encounter complex diffusing back into the reactants. Eq. 6 therefore determines the ideal relationship between chemical (F_{r_1}, $F^c_{r_2}$, $F^{nc}_{r_2}$) and diffusive-geometric (F_s) discrimination for optimal substrate catalysis flux and accuracy. However, a reaction does not need to have these specific parameters to have optimal flux and accuracy. Less stringently, if $(F^{nc}_{r_2})^{-1} \leq p \leq (F^c_{r_2})^{-1}$, the enzyme flux and accuracy will be close to optimal (32), as is the case for the ribosome (see section “Optimization of flux and accuracy” in the Supporting material).

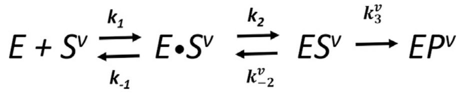

FIGURE 5 Scheme for optimization of rate and accuracy. Rates, substrates, and products are established for different substrates, v, which can correspond to correct (or cognate, [c]) or incorrect (or near-cognate, [nc]) substrates.

CONCLUSION

Enzyme promiscuity is widespread and has considerable mechanistic, evolutionary, and engineering implications (48–50). Geometric and diffusional limits on transport have direct consequences on the specificity of promiscuous enzymes as the “correct” substrate is selected from the diffusing pool of competing molecules around them. Here we have applied established kinetic expressions (30,36) to explore the diffusive-geometric effects on reaction accuracy in steady-state conditions. These results can be applied to any bimolecular reaction involving discrimination between different reactants. The basic consequence of diffusive effects on accuracy is the inclusion of geometric and solvent properties into the discrimination factors through diffusion rates (Eq. 2). To understand the role of these properties, we applied previously studied non-uniform spherical reactant geometry (24,31) as a first approximation.

Our study shows that, within different kinetic regimes, large variations arise among speed and accuracy optimization on a given geometric landscape. Importantly, even if reactions are chemically limited, accuracy can be strongly dependent on diffusive and geometric factors (see Fig. 3 *A*, right panel). Under diffusion control for a set R^{nc}_S different from R^c_S, both the speed and accuracy will increase for large R^c_S and R_E differences (Fig. 2 *B* and Fig. 3 *A* left panels). In contrast, under chemical control, the speed will be optimized for large R^c_S and R_E, while the accuracy will favor larger R^c_S over R_E (Fig. 2 *B* and Fig. 3 *A* right panels). Interestingly, for a given enzyme geometry and substrate reactivity (k^{nc}_r), the enzyme will best discriminate a specific substrate geometry (R^{nc}_S, θ^{nc}_S), as determined by Eq. 4 (see section "Accuracy for different substrate geometries").

For similar substrate geometries, the speed and accuracy are simply optimized in opposite regions of the geometric parameter (R_E, R_S) landscape (Fig. 2 *B* and Fig. 4). This speed-accuracy tradeoff falls in line with previous studies based on enzyme kinetic frameworks (5,21,51–54). Environmental parameters do not necessarily display these tradeoffs, as growing viscosity will diminish both speed and accuracy in reactions with selectivity following binding (see section “Effects of viscosity” in the Supporting material). We have also demonstrated that, in biochemically

relevant environments (such as the crowded bacterial cytoplasm), diffusive effects on accuracy can be considerable even for systems far from kinetic extremes (see section "Protein synthesis" in the Supporting material). Finally, starting from previous studies (32), we derived relations between chemical and diffusive rates that optimize the flux and accuracy (see section "Optimization of flux and accuracy" in the Supporting material).

These results explore the chemical, geometric, and diffusive landscape for a promiscuous bimolecular reaction and establish optima for its speed and accuracy. What freedom does a biological or engineered reaction possess to move around this landscape? Biochemical reactions can generally alter chemical rates of binding, recognition, and catalysis through active site mutations (16). The geometric configuration of these reactions is also malleable through the alteration of reactant surfaces (47,55), although reactant size is contingent on factors outside of speed or accuracy (56). Our results suggest that these modifications must take geometric-diffusive effects into account to control speed, specificity, or the balance between them. Inside the possibilities of evolution and engineering, the presented landscape can be navigated to achieve various decoding objectives.

In addition, these results may improve our understanding of enzyme evolution in diverse cellular and biophysical contexts. For example, recent work has shown that the density of cells systematically changes with cell size in bacteria, going from very dense cells at the small end to relatively less dense cells at the large end of bacteria (57). Since viscosity is changing with cell size, the relative size of the two terms being added in A (Eq. 4) also systematically shifts and adjusts the importance of the chemical terms of accuracy. Our prediction is that there should be a much stronger sensitivity to the chemical binding and catalytic rates in large cells compared with small cells, and this should be observable for enzymes that have evolved only in large or small cell species. This type of prediction opens new bioinformatic analyses to characterize the constraints facing the diversity of enzymes across the tree of life.

CODE AVAILABILITY

Computer code used to generate the data that support the findings of this study is available from the corresponding author upon request.

SUPPORTING MATERIAL

Supporting material can be found online at https://doi.org/10.1016/j.bpj.2022.03.005.

AUTHOR CONTRIBUTIONS

J.L.A. designed the research. J.L.A., K.P.A., and C.P.K. performed the research. J.L.A., K.P.A., and C.P.K. wrote the paper.

ACKNOWLEDGMENTS

This work was supported by NSF grant 1840301, John Templeton Foundation grant 61184, the NASA grant 80NSSC17K0295, and by HHMI Hanna Gray award to J.L.A. The authors thank Artemy Kolchinsky for helpful discussions and comments on the manuscript.

REFERENCES

1. Berg, O. P., and H. von. 1985. Diffusion-controlled macromolecular interactions. *Ann. Rev. Biophys. Biophys. Chem.* 14:131–160.
2. Schreiber, G., G. Haran, and H. X. Zhou. 2009. Fundamental aspects of protein-protein association kinetics. *Chem. Rev.* 109:839–860.
3. Bénichou, O., C. Chevalier, …, R. Voituriez. 2010. Geometry-controlled kinetics. *Nat. Chem.* 2:472–477.
4. Schavemaker, P. E., A. J. Boersma, and B. Poolman. 2018. How important is protein diffusion in prokaryotes? *Front. Mol. Biosci.* 5:1–16.
5. Tawfik, D. S. 2014. Accuracy-rate tradeoffs: how do enzymes meet demands of selectivity and catalytic efficiency? *Curr. Opin. Chem. Biol.* 21:73–80.
6. Bar-Even, A., R. Milo, …, D. S. Tawfik. 2015. The moderately efficient enzyme: futile encounters and enzyme floppiness. *Biochemistry.* 54:4969–4977.
7. Lovmar, M., and M. Ehrenberg. 2006. Rate , accuracy and cost of ribosomes in bacterial cells. *Biochimie.* 88:951–961.
8. Mellenius, H., and M. Ehrenberg. 2017. Transcriptional accuracy modeling suggests two-step proofreading by RNA polymerase. *Nucleic Acids Res.* 45:11582–11593.
9. Tsai, Y., K. A. Johnson, …, V. June. 2006. A new paradigm for DNA polymerase specificity. *Biochemistry.* 45:9675–9687.
10. Cornish-Bowden, A. 1984. Enzyme specificity: its meaning in the general case. *J. Theor. Biol.* 108:451–457.
11. Hopfield, J. J. 1974. Kinetic proofreading: a new mechanism for reducing errors in biosynthetic processes requiring high specificity. *Proc. Natl. Acad. Sci. U S A.* 71:4135–4139.
12. Ninio, J. 1974. A semi-quantitative treatment of missense and nonsense suppression in the s&A and ram ribosomal mutants of *Escherichia coli*. *J. Mol. Biol.* 84:297–313.
13. Eigen, M. 1971. Selforganization of matter and the evolution of biological macromolecules. *Naturwissenschaften.* 58:465–523.
14. Munsky, B., I. Nemenman, and B. Golan. 2009. Specificity and completion time distributions of biochemical processes. *J. Chem. Phys.* 131:235103.
15. Newton, M. S., V. L. Arcus, …, W. M. Patrick. 2018. Enzyme evolution: innovation is easy, optimization is complicated. *Curr. Opin. Struct. Biol.* 48:110–116.
16. Davidi, D., L. M. Longo, …, D. S. Tawfik. 2018. A bird's-eye view of enzyme evolution: chemical, physicochemical, and physiological considerations. *Chem. Rev.* 118:8786–8797.
17. Zhou, H., S. Wlodek, and J. Ammon. 1998. Conformation gating as a mechanism for enzyme specificity. *Proc. Natl. Acad. Sci. U S A.* 95:9280–9283.
18. Zhou, H. X. 1998. Theory of the diffusion-influenced substrate binding rate to a buried and gated active site. *J. Chem. Phys.* 108:8146–8154.
19. Fang, L., …, C. G. Zhan. 2011. Active site gating and substrate specificity of butyrylcholinesterase and acetylcholinesterase: insights from molecular dynamics simulations. *J. Phys. Chem. B.* 115:8797–8805.
20. Johnson, K. A. 2010. The kinetic and chemical mechanism of high-fidelity DNA polymerases. *Biochim. Biophys. Acta.* 1804:1041–1048.
21. Murugan, A., D. A. Huse, and S. Leibler. 2012. Speed, dissipation, and error in kinetic proofreading. *Proc. Natl. Acad. Sci.* 109:12034–12039.
22. Zaher, H. S., and R. Green. 2009. Quality control by the ribosome following peptide bond formation. *Nature.* 457:161–166.

23. Alejo, J. L., and S. C. Blanchard. 2017. Miscoding-induced stalling of substrate translocation on the bacterial ribosome. *Proc. Natl. Acad. Sci. U S A.* 114:E8603–E8610.

24. Šolc, K., and W. H. Stockmayer. 1973. Kinetics of diffusion-controlled reaction between chemically asymmetric molecules. II. Approximate steady-state solution. *Int. J. Chem. Kinet.* 5:733–752.

25. Schlosshauer, M., and D. Baker. 2004. Realistic protein-protein association rates from a simple diffusional model neglecting long-range interactions, free energy barriers, and landscape ruggedness. *Protein Sci.* 13:1660–1669.

26. Wescott, C. R., and A. M. Klibanov. 1994. The solvent dependence of enzyme specificity. *Biochim. Biophys. Acta.* 1206:1–9.

27. Gomez, D., and S. Klumpp. 2015. Biochemical reactions in crowded environments: revisiting the effects of volume exclusion with simulations. *Front. Phys.* 3:1–14.

28. Klumpp, S., M. Scott, S. Pedersen, and T. Hwa. 2013. Molecular crowding limits translation and cell growth. *Proc. Natl. Acad. Sci. U S A.* 110:16754–16759.

29. Zhou, H. 1993. Brownian dynamics study of the influences of electrostatic interaction and diffusion on protein-protein association kinetics. *Biophys. J.* 64:1711–1726.

30. Fersht, A. 1997. Structure and Mechanisms in Protein Science. W.H. Freeman and Co, New York.

31. Berg, O. G. 1985. Orientation constraints in diffusion-limited macromolecular association: the role of surface diffusion as a rate-enhancing mechanism. *Biophys. J.* 47:1–14.

32. Savir, Y., and T. Tlusty. 2013. The ribosome as an optimal decoder: a lesson in molecular recognition. *Cell.* 153:471–479.

33. Berg, O. G. 1978. On diffusion-controlled dissociation. *Chem. Phys.* 31:47–57.

34. Shoup, D., G. Lipari, and A. Szabo. 1981. Diffusion-controlled bimolecular reaction rates. *Biophys. J.* 36:697–714.

35. Johansson, M., J. Zhang, and M. Ehrenberg. 2011. Genetic code translation displays a linear trade-off between efficiency and accuracy of tRNA selection. *Proc. Natl. Acad. Sci.* 2011:1–6.

36. Fersht, A. R. 1977. Enzyme Structure and Mechanism. Freeman, New York.

37. Pavlov, M. Y., and M. Ehrenberg. 2018. Substrate-Induced formation of ribosomal decoding center for accurate and rapid genetic code translation. *Annu. Rev. Biophys.* 47:525–548.

38. Wilden, B., A. Savelsbergh, …, W. Wintermeyer. 2006. Role and timing of GTP binding and hydrolysis during EF-G-dependent tRNA translocation on the ribosome. *Proc. Natl. Acad. Sci.* U S A. 103:13670–13675.

39. Bar-even, A., E. Noor, …, R. Milo. 2011. The moderately efficient enzyme: evolutionary and physicochemical. *Biochemistry.* 50:4402–4410.

40. Kalwarczyk, T., M. Tabaka, and R. Holyst. 2012. Biologistics-Diffusion coefficients for complete proteome of *Escherichia coli*. *Bioinformatics.* 28:2971–2978.

41. Clegg, R. M. 1986. Derivation of diffusion-controlled chemical rate constants with the help of Einstein's original derivation of the diffusion constant. *J. Chem. Educ.* 63:571–574.

42. Peracchi, A. 2018. The limits of enzyme specificity and the evolution of metabolism. *Trends Biochem. Sci.* 43:984–996.

43. Rudorf, S., M. Thommen, …, R. Lipowsky. 2014. Deducing the kinetics of protein synthesis *in vivo* from the transition rates measured *in vitro*. *PLoS Comput. Biol.* 10:e1003909.

44. Schmeing, T. M., R. M. Voorhees, …, V. Ramakrishnan. 2009. The crystal structure of the ribosome bound to EF-Tu and aminoacyl-tRNA. *Science.* 326:688–694.

45. Kramer, E. B., and P. J. Farabaugh. 2007. The frequency of translational misreading errors in *E. coli* is largely determined by tRNA competition. *RNA.* 13:87–96.

46. Johansson, M., M. Lovmar, and M. Ehrenberg. 2008. Rate and accuracy of bacterial protein synthesis revisited. *Curr. Opin. Microbiol.* 11:141–147.

47. Nag, A., and A. R. Dinner. 2006. Enhancement of diffusion-controlled reaction rates by surface-induced orientational restriction. *Biophys. J.* 90:896–902.

48. Hult, K., and P. Berglund. 2007. Enzyme promiscuity: mechanism and applications. *Trends Biotechnol.* 25:231–238.

49. Khersonsky, O., and D. S. Tawfik. 2010. Enzyme promiscuity: a mechanistic and evolutionary perspective. *Annu. Rev. Biochem.* 79:471–505.

50. Leveson-Gower, R. B., C. Mayer, and G. Roelfes. 2019. The importance of catalytic promiscuity for enzyme design and evolution. *Nat. Rev. Chem.* 3:687–705.

51. Banerjee, K., A. B. Kolomeisky, and O. A. Igoshin. 2017. Elucidating interplay of speed and accuracy in biological error correction. *Proc. Natl. Acad. Sci. U. S. A.* 114:5183–5188.

52. Mallory, J. D., A. B. Kolomeisky, and O. A. Igoshin. 2019. Trade-offs between error, speed, noise, and energy dissipation in biological processes with proofreading. *J. Phys. Chem. B.* 123:4718–4725.

53. Hausser, J., A. Mayo, L. Keren, and U. Alon. 2019. Central dogma rates and the trade-off between precision and economy in gene expression. *Nat. Commun.* 10:68.

54. Pigolotti, S., and P. Sartori. 2016. Protocols for copying and proofreading in template-assisted polymerization. *J. Stat. Phys.* 162:1167–1182.

55. Andrews, S. S. 2020. Effects of surfaces and macromolecular crowding on bimolecular reaction rates. *Phys. Biol.* 17:1–23.

56. Traut, T. W. 1986. What determines the size of enzymes? *Trends Biochem. Sci.* 11:508.

57. Kempes, C. P., L. Wang, …, T. Hoehler. 2016. Evolutionary tradeoffs in cellular composition across diverse bacteria. *ISME J.* 10:2145–2157.

Biophysical Journal, Volume *121*

Supplemental information

Diffusion control in biochemical specificity

Jose L. Alejo, Christopher P. Kempes, and Katarzyna P. Adamala

Diffusion control in biochemical specificity

Jose L. Alejo, Christopher R. Kempes and Katarzyna P. Adamala

Supplementary information

Intermediate stages

If additional reversible stages leading to enzyme catalysis are present, as shown in this scheme:

$$E+S^v \underset{k_{-D}^v}{\overset{k_D^v}{\rightleftharpoons}} E\bullet S^v \underset{k_{-b}^v}{\overset{k_b^v}{\rightleftharpoons}} ES \underset{k_{-1}^v}{\overset{k_1^v}{\rightleftharpoons}} ES^{v\,1} \cdots \underset{k_{-(n-1)}^v}{\overset{k_{n-1}^v}{\rightleftharpoons}} ES^{v\,n-1} \underset{k_{-n}^v}{\overset{k_n^v}{\rightleftharpoons}} ES^{v\,n} \xrightarrow{k_{cat}^v} EP^v$$

The resulting expression for the steady-state flux increases the reactive discrimination factor according to the expression (1):

$$J^v = \frac{k_D^v[E][S]^v}{1+\frac{k_{-D}^v}{k_b^v}\left(1+\frac{k_{-b}^v}{k_1^v}\left(1+\frac{k_{-1}^v}{k_2^v}\left(1+\cdots+\frac{k_{-(n-1)}^v}{k_n^v}\left(1+\frac{k_{-n}^v}{k_{cat}^v}\right)\right)\right)\right)}$$

(S1)

Spherical geometry

The 'quasi-chemical' approximation (2) model consists of spherical reactants with reactive spherical caps (**Fig. S1**). This model involves various states ($E^{\pm}\bullet S^{\pm}$) connected by rotational motions, including a productive ($E^{+}\bullet S^{+}$) state in which the reactive patches come into contact, allowing catalysis to proceed.

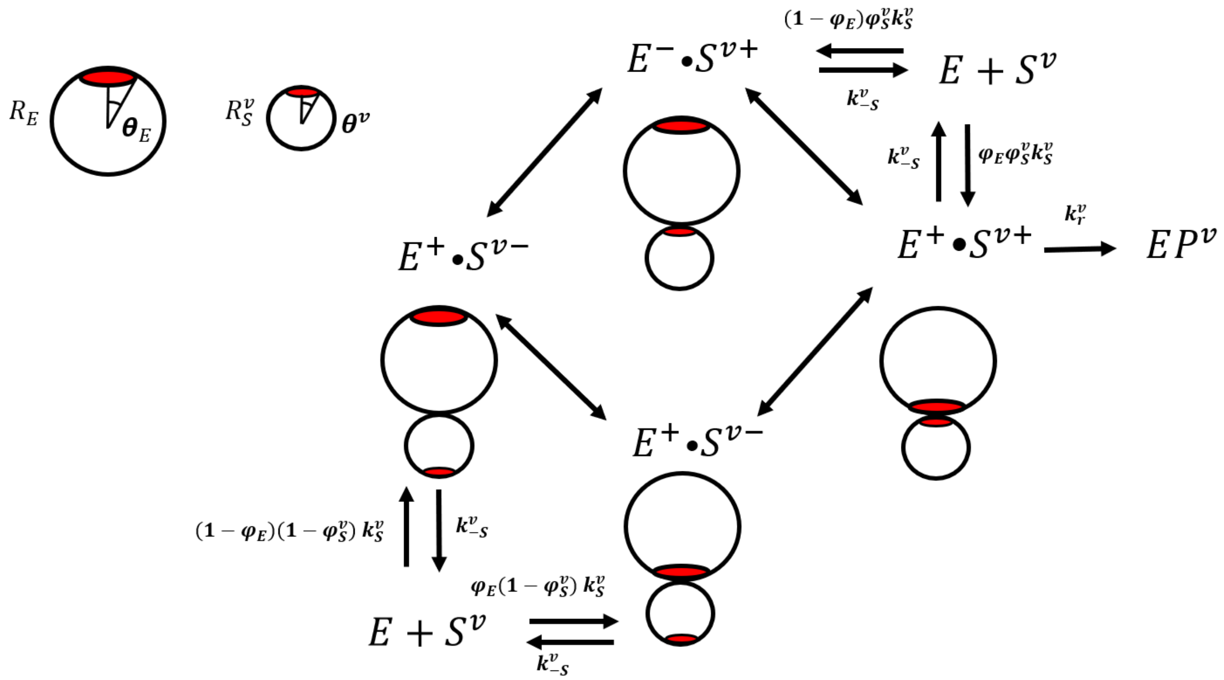

Figure S1. The quasi-chemical model for spherical reactant geometry. Reactant geometries are described by their radii and reactive surface fractions (φ), determined by the polar angles θ. The different states $E^{\pm} \bullet S^{\pm}$ can be formed with modified association constants and interconvert through rotational motions. When the reactive areas contact each other the state $E^+ \bullet S^+$ is formed and the reaction can proceed.

In the model the quantities φ are the reactive surface fractions in the interacting particles, determined by the polar angles θ. Under steady-state assumptions, the main result for the product flux is (Šolc & Stockmayer (2) Equation 1):

$$J = \frac{d[P]}{dt} = k_{eff}[E][S] = \frac{\varphi_E \varphi_S k_S [A][B]}{\Lambda_E \Lambda_S + \psi + \frac{k_{-S}}{k_r}} \tag{S2}$$

$$\psi = [(1-\Lambda_E)^{-1}(1-\Lambda_S)^{-1} + (1-\Lambda_S)^{-1}(\Lambda_E - \varphi_E)^{-1} + (1-\Lambda_E)^{-1}(\Lambda_S - \varphi_S)^{-1}]^{-1}$$

(S3)

$$\Lambda_{E,S} = \frac{\varphi_{E,S} + k_{-S}\tau_{E,S}}{1 + k_{-S}\tau_{E,S}}$$

(S4)

In their formalism, Šolc and Stockmayer established a net 'chemical' reaction rate k_r, $k_{\pm S}$ are the corresponding rates for spherical reactants and the 'reorientation parameters' $\Lambda_{E,S}$ depend on 'rotational times' (τ_E, τ_S) that are specific to each molecule. An approximation of these parameters has been obtained by comparing k_{eff} in the limit where one of the reactants is uniformly reactive, that is $\varphi_S = 1$. In this limit, $\Lambda_S = 1$ and $\psi = 0$, leading to:

$$J^* = k^*_{eff}[E][S] = \frac{\varphi k_S[E][S]}{\Lambda + \frac{k_{-S}}{k_r}}$$

(S5)

In Berg 1985 (3), this scenario was resolved exactly for spherical molecules. In this model, the reactive fraction of the surface is given by the polar angle θ, namely by the relation $\varphi = (1 - cos\theta)/2$. This gives the result (Berg 1985, Equation 17) for the effective association rate constant, k_a:

$$\frac{k_S}{k_a} = 1 + \left(\frac{1}{\varphi}\right)\left(\frac{D}{\kappa R}\right) - \frac{1}{4\varphi^2}\sum_{j=1}^{\infty}\frac{\left[P_{j-1}(cos\theta) - P_{j+1}(cos\theta)\right]^2}{(2j+1)\left[j - \xi_j K_{j+3/2}(\xi_j)/K_{j+1/2}(\xi_j)\right]}$$

(S6)

In this expression, $P_j(x)$ is the Legendre polynomial of order j and $K_j(x)$ is the modified Bessel function of the second kind of order j. Additionally, $\xi = \sqrt{j(j+1)R^2 D_R/D}$, where D_R is the rotational diffusion coefficient of particle E, $R = R_E + R_S$ is the sum of the hydrodynamic radii, $D = D_E + D_S$ is the sum of the translation diffusion coefficients and κ is the local reactivity per unit area for nonspecific association to the surface of E. By equating k^*_{eff} and k_a, the following relations are obtained:

$$\frac{k_{-S}}{k_r} = \frac{D}{\kappa R}$$

(S7)

$$\Lambda = \varphi - \frac{1}{4\varphi}\sum_{j=1}^{\infty}\frac{\left[P_{j-1}(cos\theta) - P_{j+1}(cos\theta)\right]^2}{(2j+1)\left[j - \xi_j K_{j+3/2}(\xi_j)/K_{j+1/2}(\xi_j)\right]}$$

(S8)

Much simpler approximations of Λ_C exist for diffusion-limited conditions, but this expression is general and (fairly) simple to compute numerically. This expression was used to calculate the reorientation parameters for the enzyme and substrate. This method has been performed in other studies, albeit for the diffusion-limited case (4, 5).

Applying the mentioned results to our situation, we obtain:

$$J^v = \frac{d[P]^v}{dt} = \frac{\varphi_S^v \varphi_E k_S^v [E][S]^v}{\Lambda_E^v \Lambda_S^v + \psi^v + \frac{k_{-S}^v}{k_r^v}} = \frac{\varphi_S^v \varphi_E k_S^v [E][S]^v}{\Omega^v + \frac{k_{-S}^v}{k_r^v}}$$

(S9)

$$\Omega^v = \Lambda_E^v \Lambda_S^v + \psi^v$$

(S10)

As mentioned, an exact solution of the reorientation parameters is given by the expression (3):

$$\Lambda_C = \varphi_C - \frac{1}{4\varphi_C} \sum_{j=1}^{\infty} \frac{\left[P_{j-1}(cos\theta_C) - P_{j+1}(cos\theta_C)\right]^2}{(2j+1)\left[j - \xi_{C,j} K_{j+3/2}(\xi_{C,j}) / K_{j+1/2}(\xi_{C,j})\right]}$$

(S11)

In which *C* is *E* or *S*. Using this model, we can broadly explore the effects of diffusion on enzyme accuracy. The basic geometric factors that can affect rate and accuracy are thereby radii (R_E, R_S) and the reactive fractions (φ_E, φ_S) of the molecules. To quantify these effects, the diffusion factors corresponding to spherical reactants are assigned:

$$k_S = 4\pi DR, \qquad k_{-S} = 3D/R^2, \qquad D_C = k_B T / 6\pi\eta R_C, \quad D_{C,R} = k_B T / 8\pi\eta R_C^3$$

(S12)

In these relations, *D* is the sum of the substrate and enzyme translational diffusion coefficients, which are given by D_C and *R* is the sum of the enzyme and substrate radii. These relations imply that $\xi_{S,j}^E = \sqrt{j(j+1)R^2 D_R / D} = \sqrt{j(j+1)3f^{\pm 1}(1+f^{\pm 1})/4}$, where $f = R_S/R_E$. Under these conditions, the flux is given by (following **Eq. S9**):

$$J^v = \frac{\varphi_S^v \varphi_E \left[2k_B T (R_S^v + R_E)^2 / 3\eta R_S^v R_E\right][E][S]^v}{\Omega^v + \dfrac{k_B T}{2\pi\eta(R_S^v + R_E) R_S^v R_E k_r^v}}$$

(S13)

Omega

As described by **Eqs. S10, S11 and S3**, the reorientation factor omega depends on the fraction R_S/R_E and the angles θ_E, θ_S. **Figure S2** shows heat maps of Ω as a function of R_E and R_S, for various reactant angles. In the case of similar angles, similar radii optimize the likelihood of achieving reactive site contact (**Fig. S2** left panel). In contrast, for a reactant with a larger reactive surface fraction, larger radii of this reactant and smaller radii of the other reactant are favored (**Fig. S2** middle and right panels). This difference in reactant sizes maximizes the probability of the reactive regions of the surfaces coming into contact.

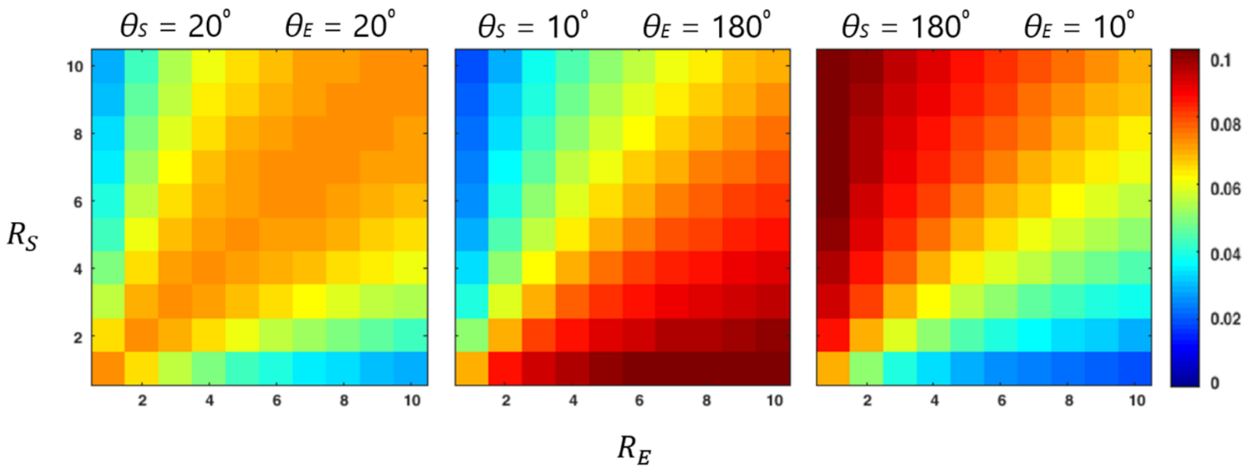

Figure S2. Dependence of the orientation factor omega (Ω) on spherical reactant geometry. Heat maps of the factor omega are shown as a function of the enzyme (R_E) and cognate substrate (R_S) radii in arbitrary length units. Maps are shown for various parameters θ_E and θ_S.

Accuracy for different geometries

As stated in the main text, when reactions are diffusion limited ($\Omega^v R_S^v R_E(R_S^v + R_E) \gg k_B T/2\pi\eta k_r^v$), the accuracy behaves as $A^{DL} = [S]^c R_S^{nc}(R_S^c + R_E)^2 \varphi_S^c \Omega^{nc}/[S]^{nc} R_S^c (R_S^{nc} + R_E)^2 \varphi_S^{nc} \Omega^c$. For enzyme sizes much larger than the substrates, the accuracy will reach the value $A_1^{DL} = [S]^c R_S^{nc} \varphi_S^c \Omega_0^{nc}/[S]^{nc} R_S^c \varphi_S^{nc} \Omega_0^c$, where the factors $\Omega_0^v = \Omega^v(f^v = R_S^v/R_E = 0)$ are independent of radii. This value is approached in the **Fig. 3a** left panel, lower right corner. On the other hand, substrates much larger than the enzyme lead to the value $A_2^{DL} =$

$[S]^c R_S^c \varphi_S^c \Omega_\infty^{nc} / [S]^{nc} R_S^{nc} \varphi_S^{nc} \Omega_\infty^c$, where $\Omega_\infty^v = \Omega^v(f^v = R_S^v / R_E = \infty)$ are independent of radii. This maximum can be seen in the **Fig. 3a** left panel, upper left corner. In the case of chemically-limited reactions ($\Omega^v R_S^v R_E (R_S^v + R_E) \ll k_B T / 2\pi\eta k_r^v$, **Fig. 3a** right panel), the accuracy behaves as $A^{CL} = [S]^c k_r^c \varphi_S^c (R_E + R_S^c)^3 / [S]^{nc} k_r^{nc} \varphi_S^{nc} (R_E + R_S^{nc})^3$. For enzyme sizes much larger than the substrates, the accuracy will reach the value $A_1^{CL} = [S]^c k_r^c \varphi_S^c / [S]^{nc} k_r^{nc} \varphi_S^{nc}$. This value is approached in the **Fig. 3a** right panel, lower right corner. Alternatively, substrates much larger than the enzyme lead to the value $A_2^{CL} = [S]^c k_r^c \varphi_S^c (R_S^c)^3 / [S]^{nc} k_r^{nc} \varphi_S^{nc} (R_S^{nc})^3$, as can be seen in **Fig. 3a** right panel, upper left corner. Finally, similar large values of the cognate substrate and the enzyme produce another local maximum, $A_3^{CL} = 8[S]^c k_r^c \varphi_S^c / [S]^{nc} k_r^{nc} \varphi_S^{nc}$, approached in the **Fig. 3a** right panel, upper right corner.

Effects of viscosity

The accuracy for different substrate geometries (**Eq. 4**) will decrease with viscosity if $\frac{\partial A}{\partial \eta} < 0$. For an accuracy of the form $A \,\alpha\, \frac{a\eta + b}{c\eta + d}$, with positive coefficients a, b, c, d, the condition $\frac{\partial A}{\partial \eta} < 0$ requires that $da < bc$. The last inequality is equivalent to $k_r^{nc} / k_{-D}^{nc} < k_r^c / k_{-D}^c$ (explicitly, $\Omega^{nc} R_S^{nc} R_E (R_S^{nc} + R_E) / \Omega^c R_S^c R_E (R_S^c + R_E) < k_r^c / k_r^{nc}$). This states that for reactions with substrate selectivity following binding, higher viscosity will decrease accuracy. This directly implies that for substrates with similar geometry (**Eq. 5**), higher viscosity will decrease accuracy if there is any chemical discrimination ($k_r^c > k_r^{nc}$).

Optimal substrate discrimination

To deduce the (near-cognate) substrate geometry that is best discriminated by an enzyme, the general expression for accuracy (**Eq. 4**) can be maximized for varying R_S^{nc}. For local maxima inside the working range of the variable ($R_S^{nc} > 0$), this is directly equivalent to maximizing $1/J^{nc}$ with respect to R_S^{nc}, with the result:

$$(R_S^{nc} + R_E)\left[(2R_S^{nc}R_E + R_E^2)\Omega^{nc} + \frac{\partial \Omega^{nc}}{\partial R_S^{nc}} R_S^{nc} R_E (R_S^{nc} + R_E)\right]$$
$$= 3\left[R_S^{nc} R_E (R_S^{nc} + R_E)\Omega^{nc} + \frac{k_B T}{2\pi\eta k_r^{nc}}\right]$$

(S14)

In the simplest case, for uniformly reactive spheres ($\theta_S^{nc} = \theta_E = 180^{\circ}$, $\Omega^{nc} = 1$), the positive solution is given by:

$$R_S^{nc*} = \left({R_E}^2 - \frac{3k_B T}{2\pi\eta R_E k_r^{nc}}\right)^{1/2}$$

(S15)

Protein synthesis

The ribosome carries out protein synthesis accurately through mRNA decoding by aminoacyl-tRNA (aatRNA). This is accomplished through codon-anticodon recognition, engagement of the codon-anticodon complex by the ribosome and a proofreading step. The 'ternary complex', composed of elongation factor Tu (EF-Tu), aatRNA (either cognate or near-cognate) and GTP (EF-Tu(GTP)-aatRNA) engages the ribosome. Complexes carrying the codon-programmed cognate aatRNA are likely activated for GTP hydrolysis on EF-Tu, while near-cognate complexes are more likely to leave the ribosome before this occurs. Additionally, after GTP hydrolysis the near-cognate complexes are similarly more likely to be released prior to peptide bond formation. In the following scheme, *E* represents the ribosome and S^v represents the cognate (or near-cognate) ternary complex:

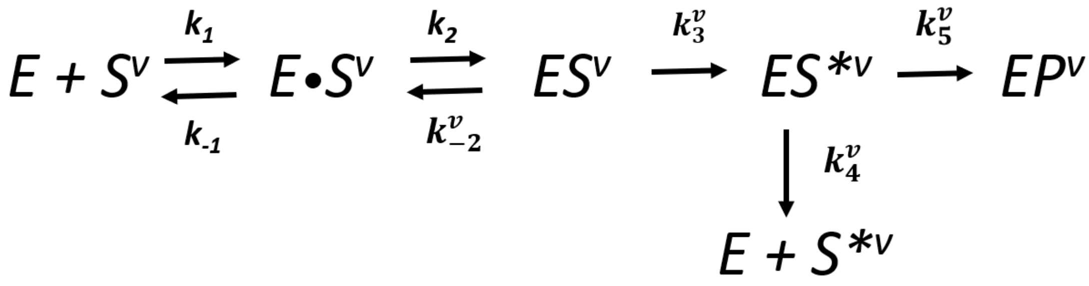

Here, the net rates of diffusive association and binding or diffusive dissociation and unbinding of the two reactants into the encounter ($E•S^v$) complex are compiled into the rates $k_1 = k_D/(1 + k_{-D}/k_b)$ or $k_{-1} = k_{-b}/(1 + k_b/k_{-D})$, respectively (5). Importantly, these rates do not encompass codon recognition, solely the initial binding/unbinding steps of the ternary complex to the ribosome. The rates k_2 and k_{-2}^v determine the codon recognition step and lead to formation of the intermediate denoted as ES^v. From this state, factor GTP hydrolysis occurs with a rate of k_3^v, leading to the formation of intermediate ES^{*v}, corresponding to the ribosome engaged to EF-Tu(GDP)-aatRNA. From this intermediate, S^{*v} can be released at a rate of k_4^v. Once the S^{*v} complex is rejected, reintegration is assumed to be negligible. Finally, the rate k_5^v is the net forward rate of peptidyl transfer leading to the cognate (or near-cognate) product, the peptide chain with the correct (or incorrect) encoded amino acid incorporated (EP^v). In this model, employing the results from the spherical quasi-chemical model, the steady-state product formation rate or product flux is given by:

$$J^v = \frac{k_S \varphi_S \varphi_E [E][S]^v}{\left[\Omega + \frac{k_{-S}}{k_b}\left(1 + \frac{k_{-b}}{k_2}\left(1 + \frac{k_{-2}^v}{k_3^v}\right)\right)\right]\left(1 + \frac{k_4^v}{k_5^v}\right)}$$

(S16)

Where k_S, k_{-S}, φ_S, φ_E and Ω are as defined in the section '**Spherical geometry**'. The accuracy is then given by (for a single near-cognate species):

$$A = \left[\frac{\frac{\Omega k_b}{k_{-D}} + 1 + \frac{k_{-b}}{k_2}\left(1 + \frac{k_{-2}^{nc}}{k_3^{nc}}\right)}{\frac{\Omega k_b}{k_{-D}} + 1 + \frac{k_{-b}}{k_2}\left(1 + \frac{k_{-2}^{c}}{k_3^{c}}\right)}\right] * \left[\frac{1 + \frac{k_4^{nc}}{k_5^{nc}}}{1 + \frac{k_4^{c}}{k_5^{c}}}\right] \frac{[S]^c}{[S]^{nc}} = I * P \frac{[S]^c}{[S]^{nc}}$$

(S17)

This expression for total accuracy is divided into accuracy achieved through the initial (*I*) and proofreading (*P*) selections. We can employ this expression to estimate translation accuracy in cells, and the effects of diffusion on this accuracy. The rates from Rudorf *et al.* (6) in **Table S1** correspond to *in vitro* protein synthesis *E. Coli* kinetic rates at 37°C. The net binding and unbinding rates κ_{on} (k_1) and ω_{off} (k_{-1}) encompass the diffusive and chemical binding steps of these reactions and are given by a specific combination of the diffusive and chemical binding rates. For the buffer conditions that these rates correspond to, we employ the environmental parameter approximation (7) $k_B T/\eta \cong 1.875\text{x}10^9 \text{M}^{-1}\text{s}^{-1}$. In the case of the *E. Coli* cell cytoplasm, viscosity for a particle with radius r_p (η_p) in this complex medium is approximated by the phenomenological expression (8):

$$\ln\left(\frac{\eta_p}{\eta_0}\right) = \left(\frac{\varepsilon^2}{R_h^2} + \frac{\varepsilon^2}{r_p^2}\right)^{-a/2}$$

(S18)

In this relation, $R_h = (42 \pm 9)\text{nm}$, $\varepsilon = (0.51 \pm 0.09)\text{nm}$ and $a = 0.53 \pm 0.04$. Using this expression, the diffusion rate k_{-D} in the cell is approximated, and **Eq. S17** is used to

calculate accuracy in the cell. The values and errors of the binding and unbinding rates were calculated using the expressions (5) $k_b = k_{-S}/[(k_S\varphi_S\varphi_E/k_1) - \Omega]$ and $k_{-b} = k_{-1}[1 + (k_b\Omega/k_{-S})]$, yielding $k_b = (2.81 \pm 0.11)\text{x}10^{10}\text{s}^{-1}$ and $k_b = (1119 \pm 105)\text{s}^{-1}$. With these values and those in **Table S1**, we obtained the values and errors of accuracies based on **Equation S17** using a custom MATLAB script.

***Rudorf et al.* source rate(s) 37°C**	***Corresponding* rate(s)**	***Units***	***Value***
κ_{on}	k_1	1/μM s	175 ± 25
ω_{off}	k_{-1}	1/s	700 ± 270
ω_{rec}	k_2	1/s	1500 ± 450
ω_{76}	k_{-2}^{nc}	1/s	1100 ± 330
ω_{78}	k_3^{nc}	1/s	7 ± 2
ω_{21}	k_{-2}^{c}	1/s	2 ± 0.6
ω_{23}	k_3^{c}	1/s	1500 ± 450
ω_{90}	k_4^{nc}	1/s	4 ± 0.7
ω_{40}	k_4^{c}	1/s	1
ω_{910}	k_5^{nc}	1/s	0.26 ± 0.04
ω_{45}	k_5^{c}	1/s	200 ± 40

Table S1. The rates from Rudorf *et al*. and the corresponding rates in the model developed in this study. The rate ω_{40} was estimated assuming it is not rate-limiting (6).

Optimization of flux and accuracy

In the study (11), a fitness function is optimized, maximizing both cognate rate and accuracy. The results are based on the maximization of the fitness function $F = J^c - dJ^{nc}$, where d represents the sensitivity of the system to errors. Any number of 'biologically reasonable' functions (for which $\partial F/\partial J^c > 0$ and $\partial F/\partial J^{nc} < 0$) are plausible. Importantly,

the parameter point obtained (**Equation 6**) will be optimal independently of the specific form of F if the condition $(F_{r_2}^{c}/F_{r_2}^{nc}) \lesssim |\partial F/\partial J^{nc}|/|\partial F/\partial J^{c}| \lesssim (F_{r_2}^{nc}/F_{r_2}^{c})$ is met (see (11) for demonstration). This condition implies that so long as J^{nc} and J^{c} are relevant to the fitness F (that is, $|\partial F/\partial J^{nc}|$ and $|\partial F/\partial J^{c}|$ are not different by too many orders of magnitude), the parameter combination in **Equation 6** will be within the optimal range for all F chosen. Though the focus of this optimization was on steady-state quantities, first-passage time and associated errors (splitting probabilities) can yield additional kinetic properties (12, 13). For the ribosome cognate and near-cognate *in vivo* discrimination factors ($F_{r_2}^{nc} \cong 160$, $F_{r_2}^{c} \cong 1/750$, $p = 0.12$, see **Table S1**), are far from the **Eq. 6** optimum ($F_{r_2}^{nc*} = 1/p^2 F_{r_2}^{c} \cong 52080$). Nonetheless, as previously calculated for *in vitro* conditions (11), the ribosome rate and accuracy is nearly optimal. To understand why this is the case, we consider that to optimize decoding the following normalized function must be maximized to 1:

$$M(\Delta_{nc}, \Delta_c) = \frac{1 + e^{(\Delta_{nc}-\Delta_c)/2}}{e^{|\Delta_c-\Delta^*|} + e^{(\Delta_{nc}-\Delta_c)/2}}$$

(S19)

Where $\Delta_v = \ln F_{r_2}^{v}$ and $\Delta^* = -\ln p - (\Delta_{nc} - \Delta_c)/2$ is the optimal near-cognate discrimination factor. This function has been calculated at 98% for *in vitro* conditions (11). For *in vivo* conditions, $\Delta_c = -6.62$, $\Delta_{nc} = 5.06$ (derived from **Table S1**, see **Protein Synthesis**). With these values, **Eq. S19** yields an optimization of ~95%. Hence, for the ribosome the flux and accuracy are optimal across a broad range of parameters. This happens because the function *M* is maximal for a 'band' of values of width $\Delta_{nc} - \Delta_c$ around the optimum $\Delta_c = \Delta^*$ (11), a parameter region equivalent to $(F_{r_2}^{nc})^{-1} \leq p \leq (F_{r_2}^{c})^{-1}$. This is intuitive since the larger this interval is, the more accurate the reaction is, and *p* can potentially grow larger, slowing down the reaction. In this way, larger accuracy permits smaller rates, keeping the

rate and accuracy optimized. While the ribosome parameters are nowhere near the optimum, they fall inside this interval, rendering the reaction's rate and accuracy highly optimized.

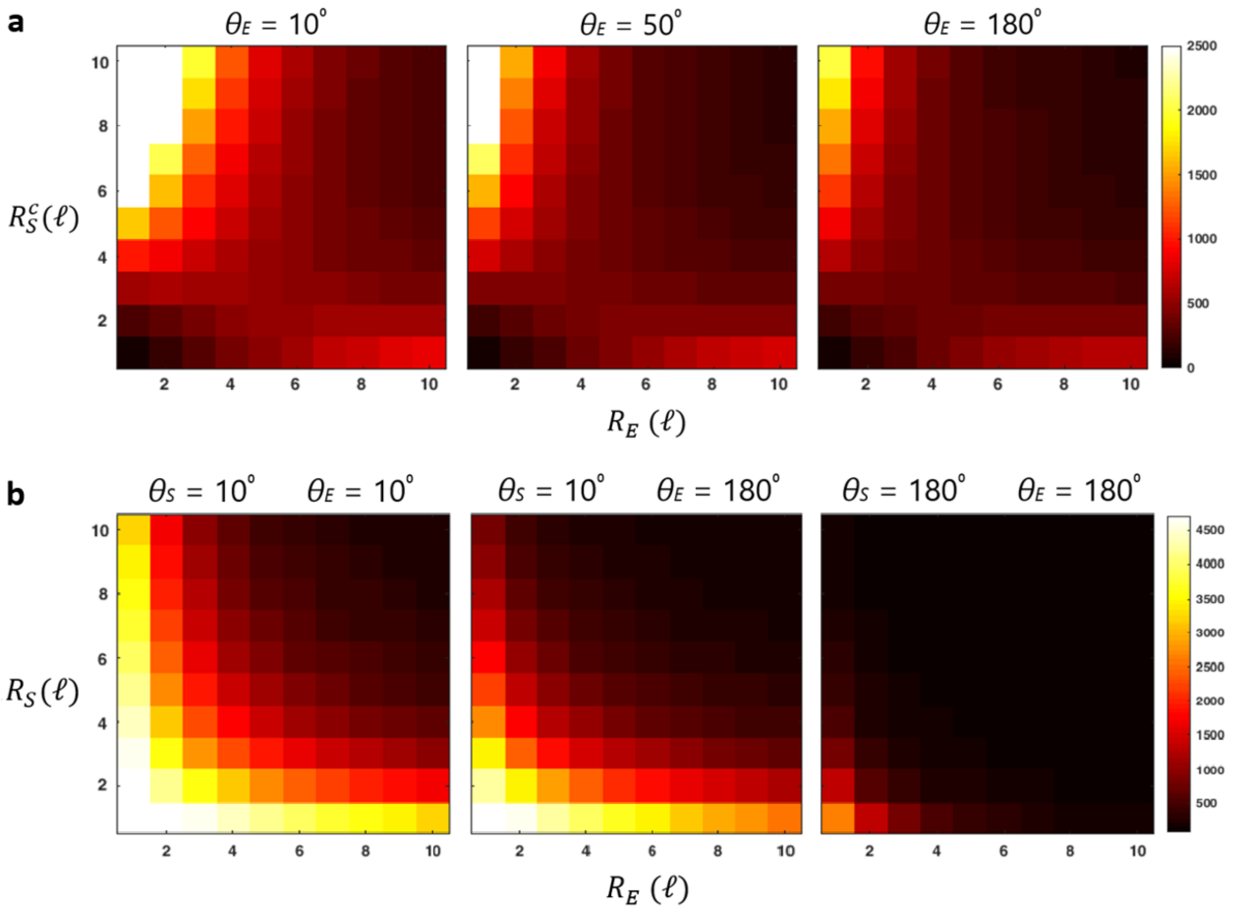

Figure S3. Dependence of accuracy on reactive surface fraction. Radii are displayed in length units $\ell = [k_B T/2\pi\eta k_0]^{1/3}$ for an arbitrary rate k_0 and $F_r^{nc}/F_r^{c} = 5000$. (**a**) Heat map of the normalized accuracy $A = (J^c/J^{nc})/([S]^c/[S]^{nc})$ for substrates with different geometries for various θ_E. The accuracy is shown as a function of the enzyme (R_E) and cognate substrate (R_S^c) radii. Parameters were set to $\theta_S^v = 10^\circ$ and $R_S^{nc} = 3\ell$. (**b**) Heat maps of the normalized accuracy for substrates with equal geometries and different θ_E and θ_S. The accuracy is shown as a function of the enzyme (R_E) and substrate (R_S) radii.

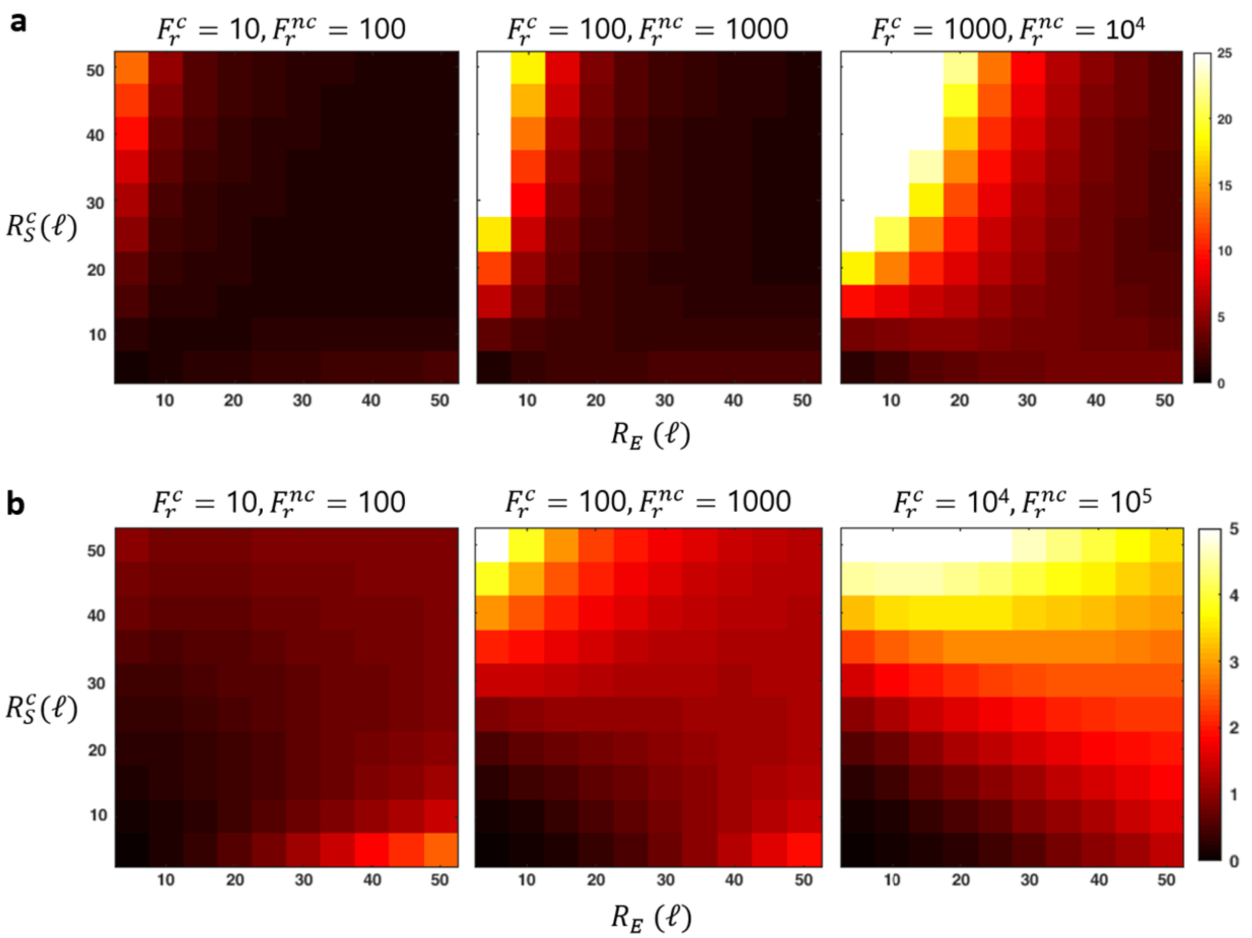

Figure S4. Dependence of accuracy on near-cognate substrate sizes. Heat maps of the normalized accuracy $A = (J^c/J^{nc})/([S]^c/[S]^{nc})$ are shown for substrates with different geometries as a function of the enzyme (R_E) and cognate substrate (R_S^c) radii in length units $\ell = [k_B T/2\pi\eta k_0]^{1/3}$ for an arbitrary rate k_0. Parameters were set to $\theta_E = 10^\circ$, $\theta_S^v = 90^\circ$. Maps are shown for different discrimination factors $F_r^v = k_0/k_r^v$, shifting from diffusion limited reactions (left panels) to chemically limited reactions (right panels). The maps correspond to near-cognate substrate sizes $R_S^{nc} = 15\ell$ (**a**) and $R_S^{nc} = 60\ell$ (**b**).

REFERENCES

1. Pavlov, M.Y., and M. Ehrenberg. 2018. Substrate-Induced Formation of Ribosomal Decoding Center for Accurate and Rapid Genetic Code Translation. *Annu. Rev. Biophys.* 47:525–548.

2. Šolc, K., and W.H. Stockmayer. 1973. Kinetics of diffusion-controlled reaction

between chemically asymmetric molecules. II. Approximate steady-state solution. *Int. J. Chem. Kinet.* 5:733–752.

3. Berg, O.G. 1985. Orientation Constraints in Diffusion-limited Macromolecular Association: The Role of Surface Diffusion as a Rate-enhancing Mechanism. *Biophys. J.* 47:1–14.

4. Zhou, H. 1993. Brownian dynamics study of the influences of electrostatic interaction and diffusion on protein-protein association kinetics. *Biophys. J.* 64:1711–1726.

5. Nag, A., and A.R. Dinner. 2006. Enhancement of diffusion-controlled reaction rates by surface-induced orientational restriction. *Biophys. J.* 90:896–902.

6. Rudorf, S., M. Thommen, M. V. Rodnina, and R. Lipowsky. 2014. Deducing the Kinetics of Protein Synthesis In Vivo from the Transition Rates Measured In Vitro. *PLoS Comput. Biol.* 10.

7. Berg, O.P. von H. 1985. Diffusion-controlled macromolecular interactions. *Ann Rev Biophys, Biophys Chem*. 14:131–60.

8. Kalwarczyk, T., M. Tabaka, and R. Holyst. 2012. Biologistics-Diffusion coefficients for complete proteome of Escherichia coli. *Bioinformatics*. 28:2971–2978.

9. Johansson, M., J. Zhang, and M. Ehrenberg. 2011. Genetic code translation displays a linear trade-off between efficiency and accuracy of tRNA selection. *Proc Natl Acad Sci*. 2011:1–6.

10. Johansson, M., and M. Lovmar. Rate and accuracy of bacterial protein synthesis revisited ˚ ns Ehrenberg. .

11. Savir, Y., and T. Tlusty. 2013. The ribosome as an optimal decoder: A lesson in molecular recognition. *Cell*. 153:471–479.

12. Banerjee, K., A.B. Kolomeisky, and O.A. Igoshin. 2017. Elucidating interplay of speed and accuracy in biological error correction. *Proc. Natl. Acad. Sci. U. S. A.* 114:5183–5188.

13. van Kampen, N.G. 2007. Stochastic processes in physics and chemistry, 3rd ed. North Holland Publishing Company.
